# Different monoclonal antibodies and immunosuppressants administration in patients with neuromyelitis optica spectrum disorder: a Bayesian network meta-analysis

**DOI:** 10.1007/s00415-023-11641-1

**Published:** 2023-03-08

**Authors:** Ziqian Yin, Youjia Qiu, Aojie Duan, Ting Fang, Zhouqing Chen, Jiang Wu, Zhong Wang, Gang Chen

**Affiliations:** 1 Department of Neurosurgery and Brain and Nerve Research Laboratory, The First Affiliated Hospital of Soochow University, 188 Shizi Street, Suzhou, 215006 Jiangsu Province China; 2 Suzhou Medical College of Soochow University, Suzhou, 215002 Jiangsu Province China; 3 Hainan General Hospital (Hainan Affiliated Hospital of Hainan Medical University), Haikou, 570311 Hainan Province China

**Keywords:** Neuromyelitis optica spectrum disorder, Relapse, RTX, AZA, Meta-analysis

## Abstract

**Background:**

A variety of novel monoclonal antibodies and immunosuppressant have been proved effective in treating Neuromyelitis Optica Spectrum Disorder (NMOSD). This network meta-analysis compared and ranked the efficacy and tolerability of currently used monoclonal antibodies and immunosuppressive agents in NMOSD.

**Methods:**

Electronic database including PubMed, Embase and Cochrane Library were searched for relevant studies evaluating monoclonal antibodies and immunosuppressants in patients with NMOSD. The primary outcome measures were annualized relapse rate (ARR), relapse rate, the Expanded Disability Status Scale (EDSS) score, and total adverse events (AEs).

**Results:**

We identified 25 studies with 2919 patients in our meta-analysis. For the primary outcome, rituximab (RTX) (SUCRA: 0.02) ranked first in reduction ARR with a significant difference compared with azathioprine (AZA) (MD – 0.34, 95% CrI – 0.55 to – 0.12) and mycophenolate mofetil (MMF) (MD –0.38, 95% CrI – 0.63 to – 0.14). Tocilizumab (SUCRA: 0.05) ranked first in relapse rate, which was superior to satralizumab (lnOR – 25.4, 95% CrI – 74.4 to – 2.49) and inebilizumab (lnOR – 24.86, 95% CrI – 73.75 to – 1.93). MMF (SUCRA: 0.27) had the fewest AEs followed by RTX (SUCRA: 0.35), both of which showed a significant difference compared with AZA and corticosteroids (MMF vs AZA: lnOR – 1.58, 95% CrI – 2.48 to – 0.68; MMF vs corticosteroids: lnOR – 1.34, 95% CrI – 2.3 to – 0.37) (RTX vs AZA: lnOR – 1.34, 95% CrI – 0.37 to – 2.3; RTX vs corticosteroids: lnOR – 2.52, 95% CrI – 0.32 to – 4.86). In EDSS score, no statistical difference was found between different interventions.

**Conclusion:**

RTX and tocilizumab showed better efficacy than traditional immunosuppressants in reducing relapse. For safety, MMF and RTX had fewer AEs. However, studies with larger sample size on newly developed monoclonal antibodies are warranted in the future.

**Supplementary Information:**

The online version contains supplementary material available at 10.1007/s00415-023-11641-1.

## Introduction

Neuromyelitis optica spectrum disorder (NMOSD) is a chronic, autoimmune-mediated, and inflammatory neurological disorder affecting the central nervous system. It has an estimated worldwide prevalence of 0.5–4.4 cases per 100,000 people [1, 2]. The resulting damage to the optic nerves, spinal cord, brain stem, and brain through inflammatory pathways [3] may cause severe motor and sensory disturbances, bladder dysfunction, vision loss, pain, and other debilitating symptoms [3, 4]. The disease course of NMOSD is recurrent rather than monophasic [5–7], and the debilitating symptoms worsen with each relapse [8]. Even one or two acute attacks can result in ambulatory disability or blindness. Therefore, preventing relapse and reducing the impact of disease-related symptoms are top priorities in NMOSD management [1].

Azathioprine (AZA), mycophenolate mofetil (MMF), and rituximab (RTX) are the most commonly used treatments for patients with NMOSD. The 2010 European Federation of Neurological Societies (EFNS) guideline recommended AZA as the first-line treatment for NMOSD [9]. However, some patients suffered from relapse and side effects after the long-term use of AZA [2]. In 80% of NMOSD cases, aquaporin 4 immunoglobulin G (AQP4-IgG) is present in astrocytic aquaporins [10]. As a pathogenic antibody of NMOSD, AQP4-IgG can cause damage and inflammation in astrocytes, eventually leading to oligodendrocyte injury and demyelination [11]. It is also commonly considered an effective target for treating neuroimmune illnesses, such as multiple sclerosis [7].

Several new biological agents with development strategies based on the mechanism of NMSOD have been evaluated in clinical trials. For example, RTX and inebilizumab [12–14] target antibody-producing B cells, eculizumab [15] targets complement protein C5, and tocilizumab [16] and satralizumab [17, 18] block interleukin-6 (IL-6) signaling. These monoclonal antibodies act on different pathways potentially involved in the pathogenesis of NMOSD. However, only one network meta-analysis (NMA), which contains no more than one monoclonal antibody medication, has been published thus far on immunosuppressants and monoclonal antibodies for the treatment of NMOSD [11]. The optimal intervention for controlling relapse and disability in NMOSD patients remains under discussion. To address this, we performed an NMA and compared the effectiveness and safety of different medications against NMOSD.

## Methods

### Study protocol

We created a study protocol following the Cochrane Collaboration framework [19]. The protocol for this meta-analysis has been retroactively filed at INPLASY (registration number: 2022120018).

### Eligibility criteria

We included studies matching the following criteria: (1) participants: adult patients (≥ 18 years) diagnosed with NMOSD according to the current and previous versions of the International Panel for Neuromyelitis Optica Diagnosis criteria [3]; (2) intervention: the monoclonal antibodies RTX, eculizumab, inebilizumab, satralizumab, and tocilizumab, the immunosuppressants AZA, MMF, cyclophosphamide, tacrolimus, and corticosteroids, and the corresponding controls (placebos); (3) outcomes: effectiveness outcomes, including the annualized rate of relapse (ARR), defined as the number of relapses divided by the time in years, the number of patients who experienced relapse, and the Expanded Disability Status Scale (EDSS) score, which is used to evaluate neurological dysfunction and disease severity in patients; safety outcomes, including total adverse events (AEs) and individual AEs most commonly reported, such as gastrointestinal intolerance, hepatotoxicity, and leukopenia (these outcomes were not necessarily present in all the included randomized controlled trials (RCTs); (4) study type: RCT, prospective and retrospective studies. We excluded studies matching at least one of the following criteria: (1) study type: conference abstracts, case reports, review articles, and noncomparative studies; (2) studies with incomplete or unreported data; (3) studies not written in English; (4) studies specifically considering patients with the myelin oligodendrocyte glycoprotein autoimmune disease (MOGAD).

### Search strategy

Two reviewers (YJQ and ZQY) searched and identified relevant studies in the electronic databases PubMed, EMBASE, and the Cochrane Library from the inception of this study to August 31, 2021. All the monoclonal antibodies and immunosuppressants used as NMOSD treatments were comprehensively searched using MeSH and Emtree terms. The complete search strategy and results are described in the supplementary material (Table S1). To ensure a comprehensive search, the RCTs included in the previous meta-analysis were also screened independently.

### Study selection and data collection

Two reviewers (ZQY and YJQ) independently examined the title, abstract, and full text of the RCTs and cohort studies obtained from three databases and matching the selection criteria. Disagreements were settled by a third author (AJD) who was not engaged in data gathering. In addition to the relevant conference abstracts for which no data was accessible, we removed research papers for which the complete text was unavailable. After the selection procedure, information from eligible studies, such as basic characteristics, inclusion and exclusion criteria, study design, and effectiveness and safety outcomes, were collected and reported in the supplementary material (Table S2).

### Quality assessment and risk of bias

Two reviewers (TF and YJQ) classified the studies by quality of evidence (high, moderate, low, or very low) using the Grading of Recommendations Assessment, Development and Evaluation (GRADE) working group approach [20]. We evaluated the risk of bias in the selected RCTs based on the Cochrane Collaboration tool using Review Manager 5.4 and assessed the bias of the included cohort studies using the Non-Randomized Studies of Interventions (ROBINS-I) tool in the R software environment 4.1.3 [21]. Two reviewers (YJQ and ZQC) classified the studies by risk of bias (low, high, or unclear) and reached a consensus through discussion. Next, we generated a funnel plot using STATA 17.0 to examine possible publication bias [22]. The asymmetric distribution of the funnel plot indicated obvious publication bias.

### Summary measures and synthesis of results

First, we merged all the dosage arms of the RCTs and cohort studies with multiple immunosuppressants and monoclonal antibody doses before including them in the analysis. We analyzed the dichotomous outcomes using the odds ratio (OR) and risk ratio (RR), while we analyzed the continuous variables using the mean difference (MD) and their respective 95% credible intervals (CrI) or confidence intervals (CI). When the data extracted from the studies described continuous variables as medians, interquartile ranges, or ranges rather than means and standard deviations, we transformed these data using the method described by Hozo et al*.* [23]. We evaluated statistical heterogeneity using the Chi-square q test and *I*^2^ statistics. *I*^2^ values < 30%, 30–50%, and > 50% indicated “low heterogeneity”, “moderate heterogeneity”, and “substantial heterogeneity,” respectively. When the heterogeneity was above 50%, we conducted a sensitivity analysis. Besides, we analyzed the data of the pairwise meta-analysis using Review Manager 5.4 software. We conducted the NMA with random effects within a Bayesian framework using the “gemtc” package of the R software environment 4.1.3 [24]. Using Stata 17.0, we produced a network graph where each node represents a drug intervention, the size of the nodes indicates the number of participants, and the thickness of the edges represents the number of trials comparing two drug therapies. We evaluated the heterogeneity in the NMA using the Chi-square q test and* I*^2^ statistics. We calculated the inconsistency by applying the node-splitting model, and* P* < 0.05 indicated significant inconsistency between direct and indirect outcomes in the NMA [25]. Besides, we produced a surface under curve ranking area (SUCRA) to report the rank probabilities of different monoclonal antibodies and immunosuppressants. For each outcome, a lower SUCRA value indicated a better intervention. We conducted detailed subgroup analyses of data from RCTs and AQP-positive groups. Using STATA 17.0, we generated a funnel plot to assess possible publication bias [22]. The asymmetric distribution of the funnel plot indicated obvious publication bias.

## Results

### Study characteristics

**Fig. 1:**
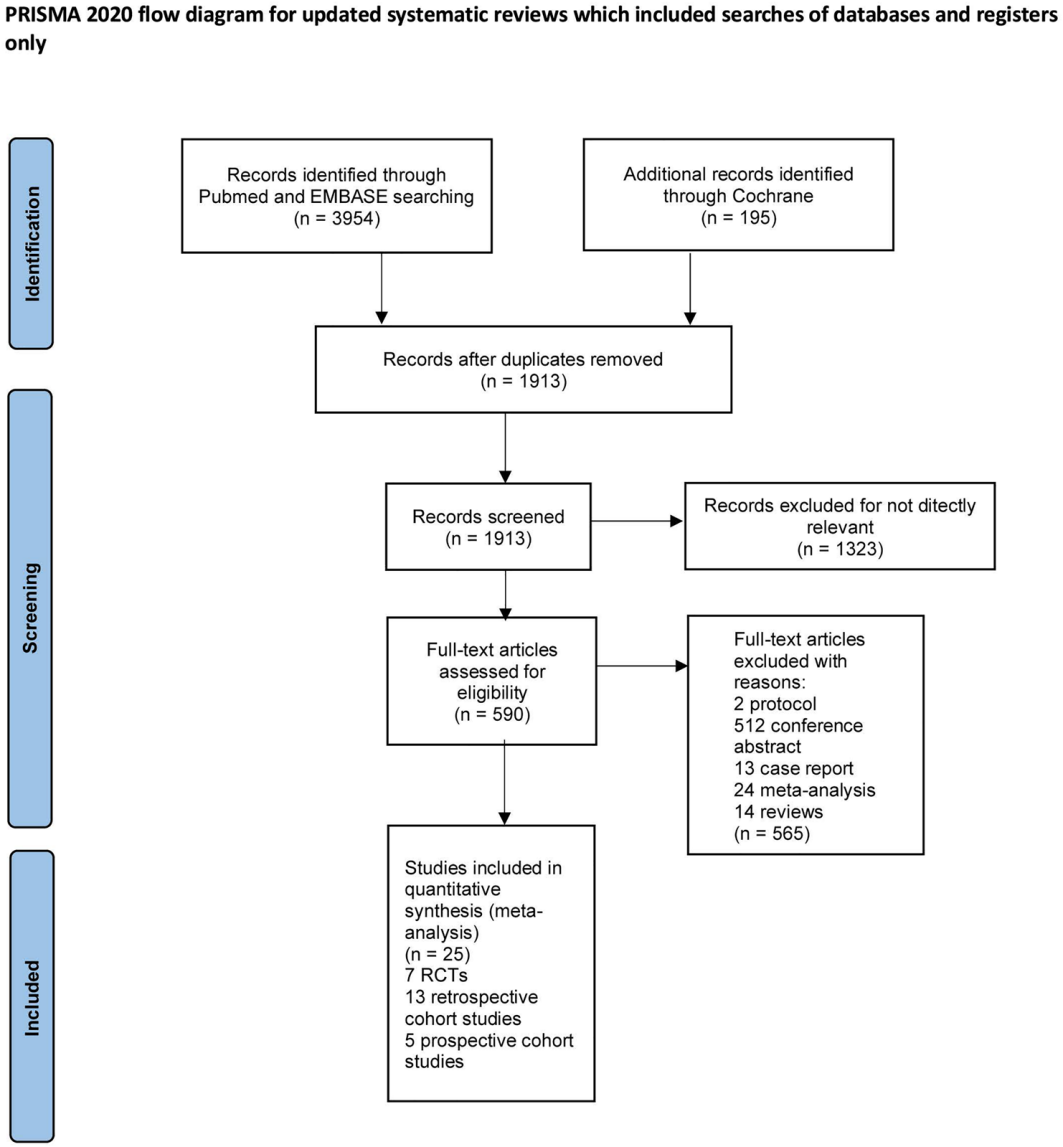
The study search, selection, and inclusion process

We combined 885 patients from five prospective cohort studies, 1259 patients from 13 retrospective cohort studies, and 775 patients from 7 RCTs. We retrieved 4149 titles and abstracts in MEDLINE, EMBASE, and the Cochrane Library. We excluded 2,236 articles due to duplication. The quick review eliminated a total of 1323 irrelevant articles. Next, we assessed the eligibility of 590 full articles. The final 565 retained studies were 24 meta-analyses, 2 protocols, 14 reviews, 13 case reports, and 512 conference abstracts. Figure 1 displays the flowchart and basic characteristics of the seven included RCTs outlined in Table 1. Ten different drugs were compared with a placebo or each other, namely AZA, RTX, tocilizumab, eculizumab, satralizumab, inebilizumab, MMF, tacrolimus, cyclophosphamide, and corticosteroids (Tables 2, 3).

**Table 1:** Characteristics of the included randomized controlled trials for patients

Study	Year	Countries	Centers	Study period (months)	Outcome events	Treatment group, (no. of participants)	Male (%)	Mean age ± SD (years)
Nikko et al.	2017	Iran	1	15	EDSS, ARR, relapse, AEs, gastrointestinal intolerance, hepatotoxicity	Azathioprine	20	32.35 ± 9.56
						Rituximab	12.1	35.33 ± 8.98
Zhang et al.	2020	China	6	15	EDSS, relapse, AEs, gastrointestinal intolerance, hepatotoxicity	Azathioprine	10.2	45.30 ± 14.50
						Tocilizumab	6.8	48.10 ± 13.40
Pittock et al.	2019	Global	70	12	EDSS, ARR, AEs, gastrointestinal intolerance	Eculizumab	8.3	43.19 ± 13.30
						PLA	10.6	45.00 ± 13.30
Tahara et al.	2020	Japan	8	24	EDSS, ARR, relapse, AEs, gastrointestinal intolerance	Rituximab	10.5	43.48 ± 21.63
						PLA	0	44.28 ± 20.83
Yamaura et al.	2019	Global	141	6	EDSS, ARR, relapse, AEs	Satralizumab	9.8	35.40 ± 16.90
						PLA	4.8	38.80 ± 12.00
Traboulsee et al.	2020	Global	44	18	EDSS, ARR, relapse, AEs, gastrointestinal intolerance	Satralizumab	27	36.40 ± 10.70
						PLA	3.1	39.30 ± 13.30
Cree et al.	2019	Global	99	6	Relapse, AEs, gastrointestinal intolerance	Inebilizumab	8.6	43.00 ± 11.60
						PLA	10.7	42.60 ± 13.90

*PLA* placebo; *ARR* annualized rate of relapse; *AEs* adverse events; *EDSS* the Expanded Disability Status Scale

**Table 2:** Summary and detailed effects sizes of different monoclonal antibodies and immunosuppressants from pairwise meta-analysis of safety outcomes

Safety outcomes or subgroup title (total and by drug)	No. of trials contributing to the meta-analysis	No. of participants contributing to the meta-analysis	OR [95% CI]	*P* value	*I*^2^ (%)	GRADE
AEs						
vs Placebo	5	589	1.18 [0.63, 2.20]	0.61	24	Low^a,b^
Eculizumab	1	143	1.02 [0.29, 3.59]	0.97	N/A	High
Rituximab	1	38	1.00 [0.13, 7.94]	1.00	N/A	High
Satralizumab	2	178	1.48 [0.19, 11.72]	0.71	74	Moderate ^b^
Inebilizumab	1	230	0.93 [0.47, 1.84]	0.84	N/A	High
vs Corticosteroids	1	239	0.13 [0.01, 2.03]	0.15	92	Very low^a^
Azathioprine	1	160	0.51 [0.23, 1.10]	0.09	N/A	Low
Mycophenolate mofetil	1	79	0.03 [0.01, 0.12]	< 0.00001	N/A	Low
Gastrointestinal intolerance						
vs Placebo	4	506	0.63 [0.28, 1.39]	0.25	47	Low^a,b^
Eculizumab	1	143	0.55 [0.27, 1.12]	0.10	N/A	High
Rituximab	1	38	0.21 [0.02, 2.07]	0.18	N/A	High
Satralizumab	1	95	3.17 [0.66, 15.29]	0.15	N/A	Moderate ^b^
Inebilizumab	1	230	0.43 [0.18, 1.03]	0.06	N/A	High
vs Corticosteroids	1	239	0.34 [0.04, 2.85]	0.32	0	Very low^a,e^
Azathioprine	1	160	0.34 [0.02, 5.55]	0.45	N/A	Low
Mycophenolate mofetil	1	79	0.35 [0.01, 8.87]	0.52	N/A	Low
Hepatotoxicity						
vs Corticosteroids	1	239	0.74 [0.07, 8.08]	0.81	57	Very low^a,e^
Azathioprine	1	160	1.83 [0.50, 6.66]	0.36	N/A	Low
Mycophenolate mofetil	1	79	0.14 [0.01, 2.86]	0.20	N/A	Low
Leukopenia						
vs Corticosteroids	1	239	0.30 [0.03, 2.65]	0.28	57	Very low^a,e^
Azathioprine	1	160	0.65 [0.24, 1.74]	0.39	N/A	Low
Mycophenolate mofetil	1	79	0.06 [0.00, 1.09]	0.06	N/A	Low

*OR* odds ratio; *CI* confidence interval; *AEs* adverse events

^a^Indirectness

^b^Limitations (risk of bias)

^c^Imprecision

^d^Inconsistency

^e^Publication bias

**Table 3:** Summary and detailed effects sizes of different monoclonal antibodies and immunosuppressants from pairwise meta-analysis of seven efficacy outcomes

Outcomes (total and by drug)	No. of trials contributing to the meta-analysis	No. of participants contributing to the meta-analysis	MD (95% CI)/OR [95% CI]	*P* value		*I*^2^ (%)	GRADE
ARR							
vs Placebo	4	359	− 0.31 (− 0.53, − 0.08)	0.007	55		Low^a,b^
Eculizumab	1	143	− 0.20 (− 0.51, 0.11)	0.21	N/A		High
Rituximab	1	38	− 0.96 (− 1.54, − 0.38)	0.001	N/A		High
Satralizumab	2	178	− 0.25 (− 0.41, − 0.09)	0.002	4		Moderate^b^
vs Corticosteroids	1	239	− 0.25 (− 0.53, 0.04)	0.09	0		Very low^a,e^
Azathioprine	1	160	− 0.20 (− 0.59, 0.19)	0.31	N/A		Low
Mycophenolate mofetil	1	79	− 0.30 (− 0.73, 0.13)	0.17	N/A		Low
Relapse							
vs Placebo	5	589	0.19 [0.09, 0.42]	< 0.0001	61		Low^a,b^
Eculizumab	1	143	0.04 [0.01, 0.16]	< 0.00001	N/A		High
Rituximab	1	38	0.04 [0.00, 0.82]	0.04	N/A		High
Satralizumab	2	178	0.38 [0.20, 0.73]	0.004	0		Moderate^b^
Inebilizumab	1	230	0.21 [0.10, 0.43]	< 0.0001	N/A		High
vs Corticosteroids	2	313	0.36 [0.21, 0.61]	0.0002	0		Very low^a^
Tocilizumab	1	41	0.19 [0.04, 0.83]	0.03	N/A		Low
Azathioprine	1	90	0.48 [0.20, 1.16]	0.10	N/A		Low
Mycophenolate mofetil	1	182	0.33 [0.15, 0.73]	0.006	N/A		Low
EDSS							
vs Placebo	4	359	− 0.17 (− 0.35, 0.01)	0.07	0		Low^a,b^
Eculizumab	1	143	− 0.30 (− 0.62, 0.02)	0.06	N/A		High
Rituximab	1	38	− 0.08 (− 0.35, 0.19)	0.57	N/A		High
Satralizumab	2	178	− 0.15 (− 0.50, 0.20)	0.41	0		Moderate^b^
vs Corticosteroids	1	239	− 0.69 (− 1.19, − 0.18)	0.007	0		Very low^a,e^
Azathioprine	1	160	− 0.50 (− 1.14, 0.14)	0.12	N/A		Low
Mycophenolate mofetil	1	79	− 1.00 (− 1.82, − 0.18)	0.02	N/A		Low

*MD* mean difference; *OR* odds ratio; *CI* confidence interval; *ARR* annualized rate of relapse; *EDSS* expanded disability status scale

^a^Indirectness

^b^Limitations (risk of bias)

^c^Imprecision

^d^Inconsistency

^e^Publication bias

### Network meta-analysis of all outcomes

**Fig. 2:**
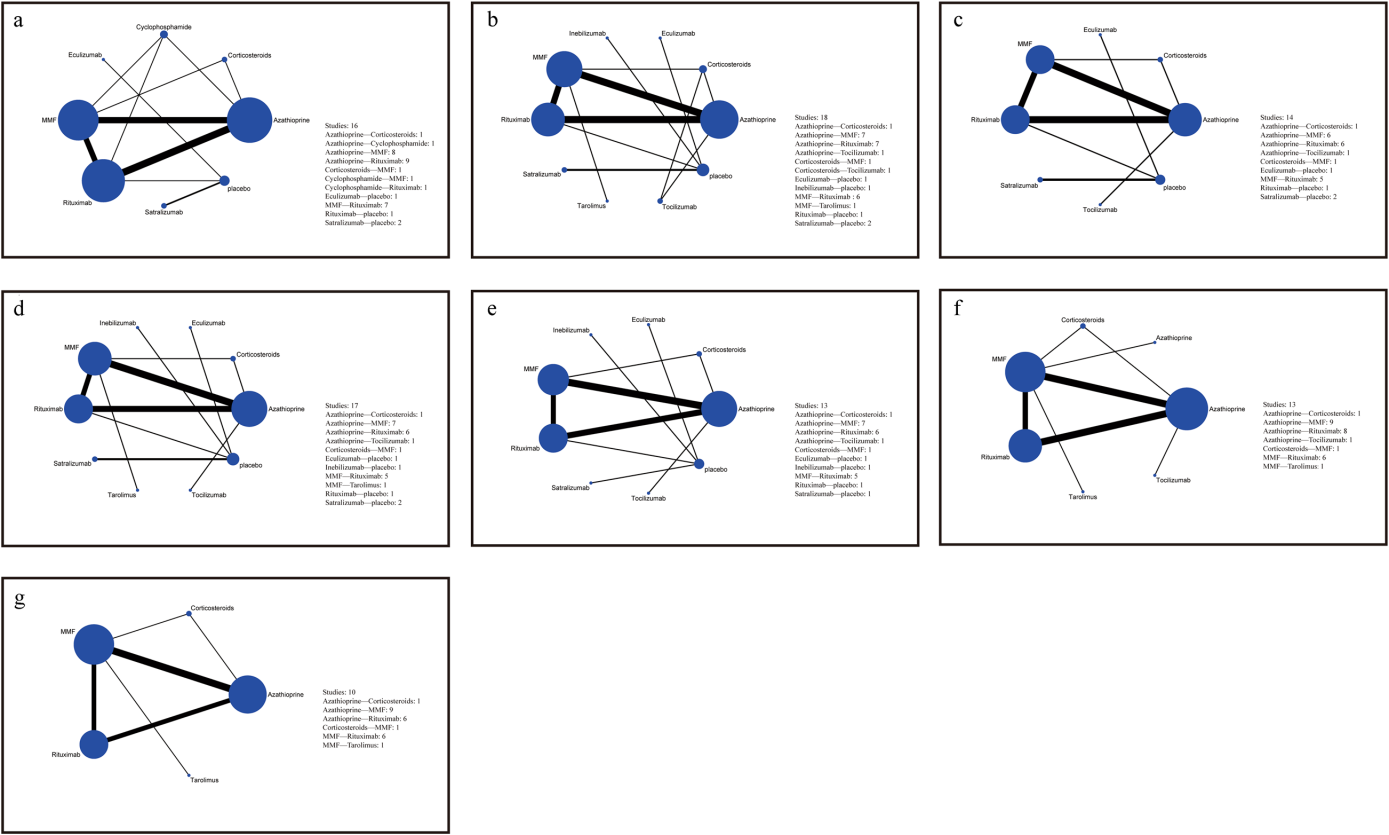
Network of trails comparing different monoclonal antibodies and immunosuppressants of NMOSD treatments. The size of circles represented the number of participants for each intervention and the width of lines represented the number of trials compared between treatments. **a** ARR. **b** Relapse. **c** EDSS score. **d** Total adverse Events. **e** Gastrointestinal intolerance **f** Hepatotoxicity. **g** Leukopenia

**Fig. 3:**
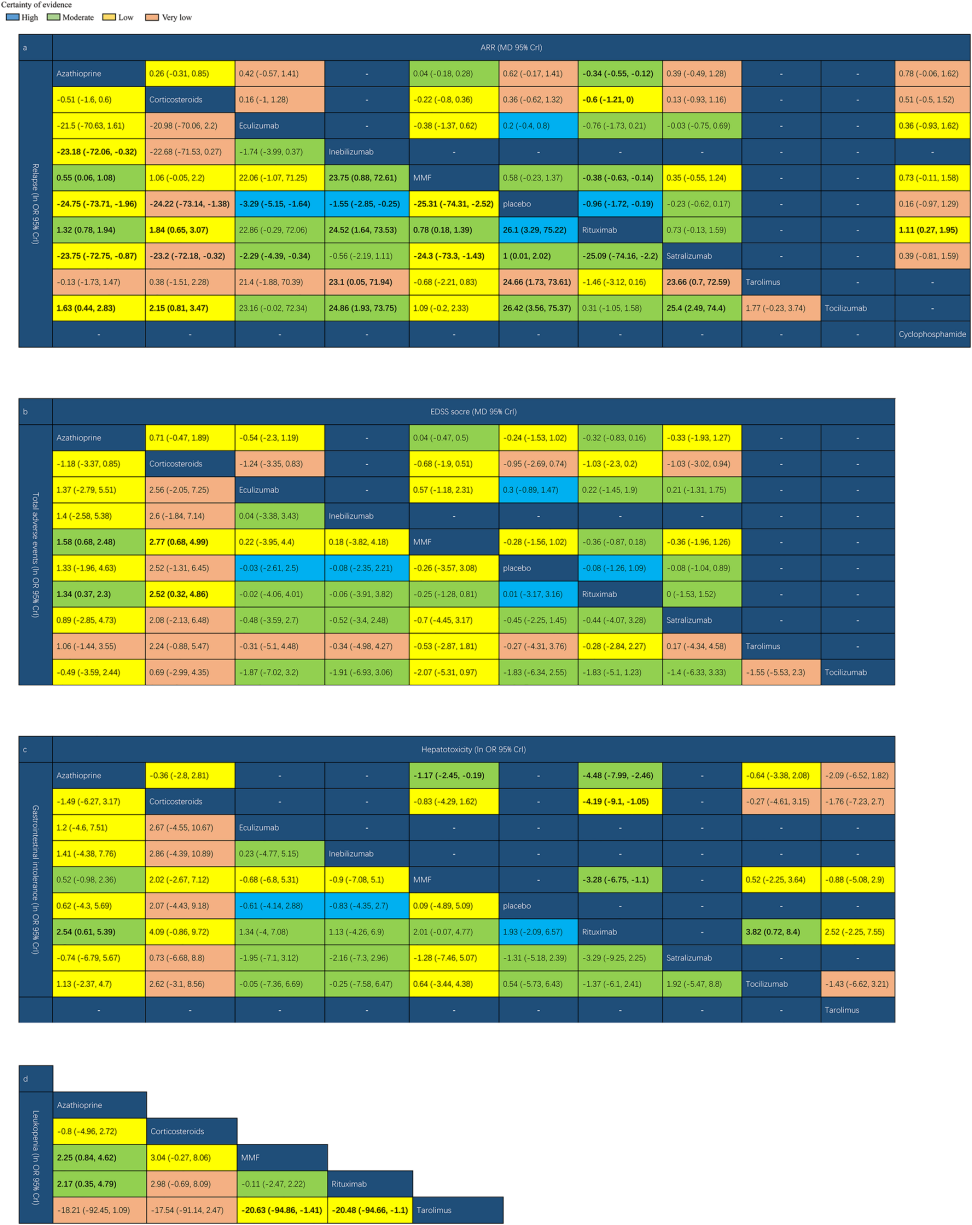
Network meta-analysis results of NMOSD treatments. **a** ARR and relapse. **b** EDSS score and total adverse events. **c** Gastrointestinal intolerance and hepatotoxicity. **d** Leukopenia. Values in bold indicate significant difference

The network graph for different interventions appears in Fig. 2 and the forest plot of NMA was illustrated in Figs. S3–S9. In terms of efficacy, RTX was associated with a lower ARR than MMF, cyclophosphamide, corticosteroids, AZA, and placebo (MD − 0.96, 95%CrI − 1.72 to − 0.19) (Fig. 3a). Patient groups treated with AZA (lnOR − 24.75, 95%CrI − 73.71 to − 1.96), eculizumab (lnOR − 3.29, 95%CrI − 5.15 to − 1.64), inebilizumab (lnOR − 1.55, 95% CrI − 2.85 to − 0.25), MMF (lnOR − 25.31, 95% CrI − 74.31 to − 2.52), RTX (lnOR − 26.1, 95%CrI − 75.22 to − 3.29), satralizumab (lnOR − 1, 95%CrI − 2.02 to − 0.01), tacrolimus (lnOR − 24.66, 95% CrI − 73.61 to − 1.73), and tocilizumab (lnOR − 26.42, 95% CrI − 75.37 to − 3.56) had lower relapse rates than placebo groups (Fig. 3a). However, we found no statistically significant differences between the EDSS scores associated with these treatments and placebo (Fig. 3b).

In terms of safety, MMF was associated with a lower incidence of total AEs than AZA (lnOR − 1.58, 95% CrI − 2.48 to − 0.68) and corticosteroids (lnOR − 2.77, 95% CrI − 4.99 to − 0.68) (Fig. 3b). The incidence of gastrointestinal intolerance was lower with RTX than with AZA (lnOR − 2.54, 95% CrI − 5.39 to − 0.61) (Fig. 3c). AZA (lnOR 4.48, 95% CrI 2.46 to 7.99), MMF (lnOR 3.28, 95% CrI 1.1 to 6.75), and tocilizumab (lnOR 3.82, 95% CrI 0.72 to 8.4) exhibited a higher incidence of hepatotoxicity than RTX (Fig. 3c). Finally, compared with tacrolimus, MMF (lnOR − 20.63, 95% CrI − 94.86 to − 1.41) and RTX (lnOR − 20.48, 95%CrI − 94.66 to − 1.1) were associated with lower leukopenia incidences (Fig. 3d).

### Pairwise meta-analysis of all outcomes

In terms of efficacy outcomes, RTX (MD − 0.96, 95% CI − 1.54 to − 0.38, *I*^*2*^ N/A, *P* < 0.01, high certainty evidence) and satralizumab (MD − 0.25, 95% CI − 0.41 to − 0.09, *I*^*2*^ 4, *P* < 0.01, moderate certainty evidence) reduced ARR more efficiently than a placebo. Eculizumab (OR 0.04, 95% CI 0.01 to 0.16, *I*^*2*^ N/A, *P* < 0.01, high certainty evidence), satralizumab (OR 0.38, 95% CI 0.20 to 0.73, *I*^*2*^ 0, *P* < 0.01, moderate certainty evidence) and inebilizumab (OR 0.21, 95% CI 0.10 to 0.43, *I*^*2*^ N/A, *P* < 0.01, high certainty evidence) reduced recurrence more effectively than a placebo. Additionally, MMF (OR 0.33, 95% CI 0.15 to 0.73, *I*^*2*^ N/A, *P* < 0.01, low certainty evidence) reduced recurrence more effectively than corticosteroids. Moreover, MMF (MD − 1.00, 95% CI − 1.82 to − 0.18, *I*^*2*^ N/A, *P* 0.02, low certainty evidence) reduced the EDSS score more effectively than corticosteroids. The detailed results are shown in Figs. S27–S32.

In terms of safety, we found that monoclonal antibodies and immunosuppressants did not cause a statistically higher incidence of AEs than placebos and corticosteroids, and MMF was associated with a lower incidence of total AEs than corticosteroids (OR 0.03 95% CI 0.01 to 0.12, *I*^*2*^ N/A, *P* < 0.01). The detailed results are shown in Figs. S33–S38. We also performed sensitivity analyses for ARR, total AEs, hepatotoxicity, and leukopenia, and all statistics were robust (Figs. S39–S43).

### Rank probability

**Fig. 4:**
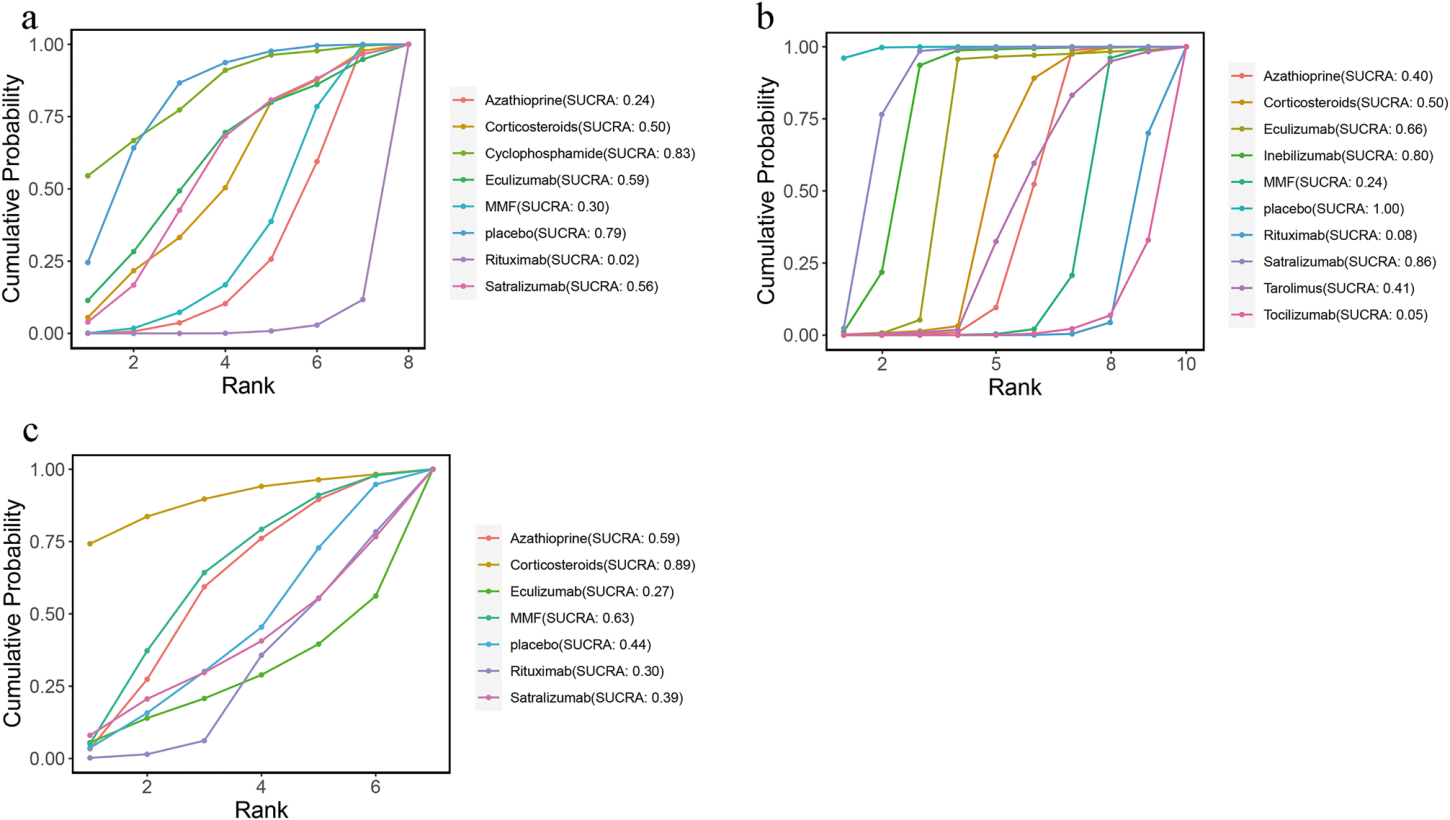
Cumulative probability of each intervention for efficacy outcomes. A smaller SUCRA value indicated a better rank for the intervention. **a** ARR. **b** Relapse. **c** EDSS score

**Fig. 5:**
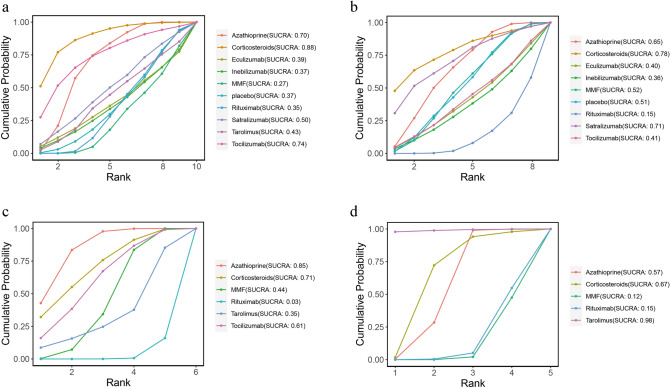
Cumulative probability of each intervention for safety outcomes. A smaller SUCRA value indicated a better rank for the intervention. **a** Total adverse events. **b** Gastrointestinal intolerance **c** Hepatotoxicity. **d** Leukopenia

The ranking probability of different monoclonal antibodies and immunosuppressants in patients with NMOSD appears in Figs. 4 and 5. According to the SUCRA plot and values, RTX (SUCRA, 0.019) ranked the highest in ARR, while cyclophosphamide (SUCRA, 0.826) ranked the lowest (Fig. 4a). Regarding relapse rates, tocilizumab (SUCRA, 0.047) ranked the highest, while placebo (SUCRA, 0.995) ranked the lowest (Fig. 4b). Eculizumab (SUCRA, 0.269) ranked the highest in EDSS score, while corticosteroids (SUCRA, 0.895) ranked the lowest (Fig. 4c). In terms of total AEs, MMF (SUCRA, 0.269) showed the lowest incidence of total AEs, whereas corticosteroids (SUCRA, 0.884) had the highest incidence (Fig. 5a). For separated AEs, RTX (SUCRA, 0.146) had the lowest incidence of gastrointestinal reactions, while corticosteroids (SUCRA, 0.785) had the highest incidence (Fig. 5b). RTX (SUCRA, 0.032) and AZA (SUCRA, 0.846) had the lowest and highest incidences of hepatotoxicity, respectively (Fig. 5c). Finally, MMF (SUCRA, 0.123) had the highest leukopenia incidence, while tacrolimus (SUCRA, 0.978) showed the highest (Fig. 5d).

### Heterogeneity and consistency analysis

The network *I*^2^ values for the three efficacy and four safety outcomes are shown in Figs. S10–S16. For three networks with an *I*^*2*^ value over 50% (namely ARR, relapse, and EDSS), we employed a node-split model to compare the consistency and inconsistency of direct and indirect comparisons. As illustrated in Figs. S17–S19, we found no significant inconsistency in the network model, indicating that the results were relatively reliable.

### Subgroup analyses of RCTs and AQP-positive groups

We performed detailed subgroup analyses of data from RCTs (*n = *7) (Figs. S44–S57). In terms of efficacy, tocilizumab (SUCRA: 0.07) was associated with a lower recurrence rate than the placebo (lnRR − 30.67, 95% CrI − 93.27 to − 3.97), satralizumab (lnRR − 30.04, 95% CrI − 92.55 to − 3.26), and inebilizumab (lnRR − 29.51, 95% CrI − 92.2 to − 2.63). There were no statistically significant differences in ARR and EDSS between these groups. In terms of safety, we found no significant statistical differences between the various treatments (Fig. S45). The network heterogeneity analyses are shown in Figs. S53–S57.

We also performed a detailed subgroup analysis of AQP4-IgG seropositive patients from RCTs (*n = *6) (Figs. S58–S62). In terms of efficacy, RTX is better than a placebo (MD − 0.96, 95%CI − 1.54 to − 0.38) for reducing ARR (Fig. S58). Eculizumab, satralizumab, and inebilizumab are associated with lower recurrence rates than a placebo, and tocilizumab was associated with lower relapse rates than AZA (RR 0.25, 95% CI 0.11 to 0.57) (Fig. S59). In terms of safety, there were no significant statistical differences between the various treatment modalities (Figs. S61–S62).

### Risk of bias in included studies and publication bias

**Fig. 6:**
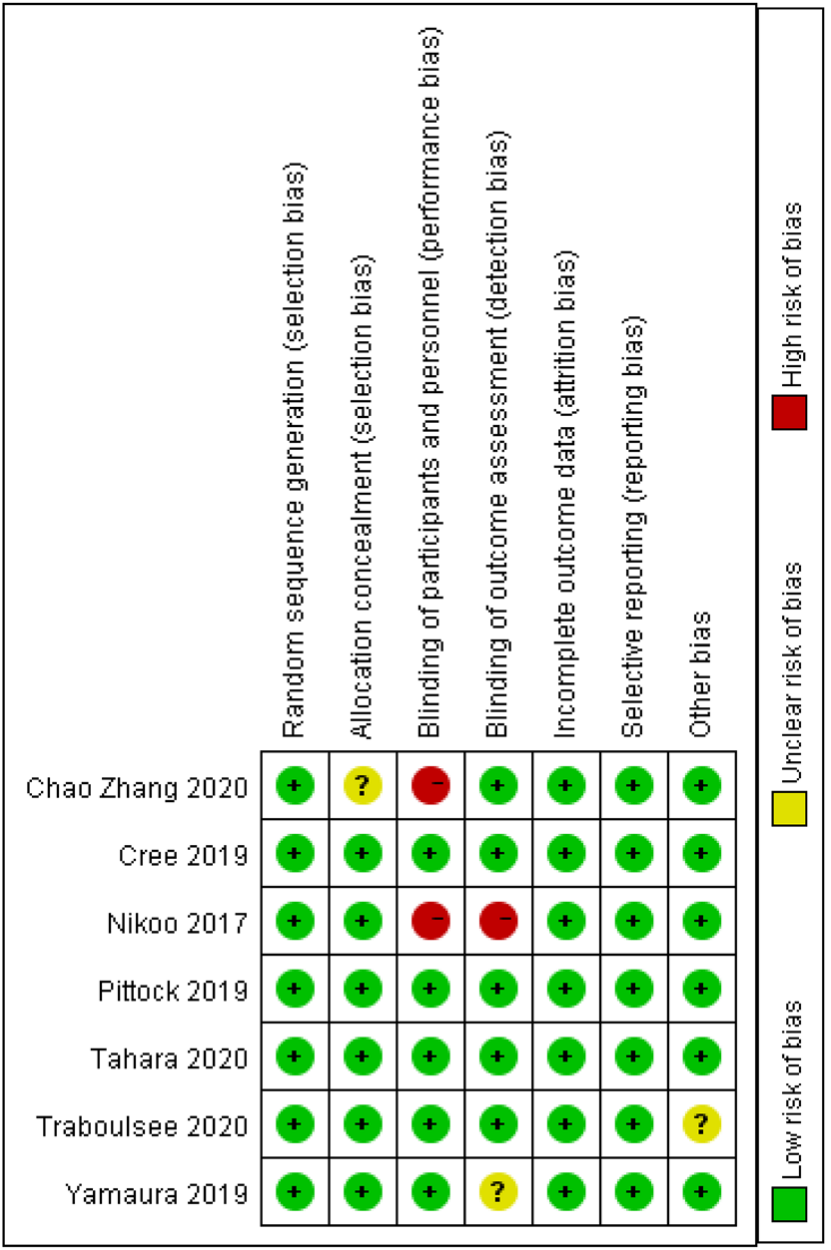
Risk of bias: a summary table for each risk of bias item for each study

The risk of bias for the included RCTs is shown in Fig. 6. We observed a low risk of bias for random sequence generation in all of the included RCTs. One RCT revealed an unclear bias risk for allocation concealment. In terms of participant and staff blinding as well as outcome assessment blinding, three RCTs had a high risk of bias, and one had an unclear risk of bias. An unclear risk of bias on selective reporting was reported in one study. In addition to these factors, one RCT showed an unclear risk of bias. We also assessed the risk of bias for cohort studies using the ROBINS-I method (Figs. S1–S2). The funnel plot was relatively symmetric, indicating the absence of publication bias among the original studies potentially affecting the NMA (Figs. S20–S26) [26].

## Discussion

NMOSD is a rare inflammatory neurological disease that induces permanent disability and recurrent symptoms in patients. Some medications, developed based on the etiology and pathology of NMOSD, have been approved for treating it. However, the real-world efficacy and safety of the various NMSOD treatments remain unclear. We performed an NMA on currently used monoclonal antibodies and immunosuppressant for the treatment of NMOSD. Besides, we conducted subgroup analyses of RCTs to obtain comprehensive high-quality results. Considering the key role of IL-6 in the activation of B cells through AQP4-IgG in NMOSD, we also performed a subgroup analysis of medications in AQP4-IgG seropositive patients [27]. Our study may help clinicians gain a more comprehensive understanding of the treatment of NMOSD, helping them make better clinical decisions.

Because the progress of NMOSD is strongly correlated with the number of recurrences and may cause permanent disability, the primary purpose of treatment is to prevent recurrence. The ARR has been widely used to evaluate the risk of recurrence in NMOSD-related systematic reviews and clinical trials [28]. Combined with the analysis of relapse rates, the effect of a specific monoclonal antibody or immunosuppressant on reducing recurrence in NMOSD patients is comprehensively evaluated. According to our cumulative probability ranking, RTX was more effective than other monoclonal antibodies or immunosuppressants in lowering ARR, which is consistent with the results obtained by Nikoo et al. [12]. RTX is a monoclonal antibody against CD20 in B cells [29] that depletes B cells in peripheral blood and was thus initially approved for treating B cell lymphoma [13]. AQP4-IgG has been widely recognized as a potential target for treating neuroimmune diseases, including NMOSD, which justifies the use of therapies targeting antibody-producing B cells [7, 30]. Several open-label studies have proven the ability of RTX to prevent recurrence [31–33]. A retrospective study conducted by Mealy et al. demonstrated that RTX and MMF were superior to AZA in reducing ARR. Regarding relapse rates, tocilizumab yielded significantly superior outcomes than satralizumab, inebilizumab, and AZA [2]. In 2020, an RCT conducted by Zhang et al. showed that tocilizumab significantly and effectively reduced the number of patients suffering relapses [16]. Clinical studies have indicated that IL-6 plays a critical role in the pathogenesis of NMOSD [34, 35]. As the first humanized IL-6 receptor monoclonal antibody, tocilizumab has been approved to treat various autoimmune diseases [36]. Uzawa et al. confirmed that tocilizumab was appropriate for treating NOMSD by showing that NMSOD patients had relatively higher IL-6 levels in the cerebrospinal fluid during relapse [33, 37]. Additionally, several retrospective studies demonstrated that tocilizumab effectively reduced the relapse rate in patients with NMOSD [38–40], including those who were irresponsive to immunosuppressive drugs or RTX. Our subgroup analyses of RCTs also confirmed the efficacy of RTX and tocilizumab in reducing relapse in NMOSD patients. In AQP4-IgG-positive patients, the majority of monoclonal antibodies reduced relapse rates more effectively than placebos and immunosuppressants. Moreover, RTX and tocilizumab were the most effective treatments for preventing recurrence in NMOSD patients, and most patients with NMOSD were highly responsive to long-term RTX interventions, especially AQP4-IgG-positive patients. Tocilizumab was more suitable for NMOSD patients with an invalid response after RTX treatment [41].

The EDSS was initially developed for evaluating neurological deficits in patients with multiple sclerosis, and has been used for the clinical assessment of patients with NMOSD [42]. According to the SUCRA outcome, eculizumab ranked first in improving neurological deficits in NMOSD patients, albeit without statistical significance compared with other medications. Eculizumab is a humanized monoclonal antibody that prevents the cleavage of the terminal complement protein C5 into C5a and C5b [43]. The FDA approved it for the treatment of AQP4-IgG-positive adult patients with NMOSD [44]. Pittock et al. performed an RCT showing that eculizumab did not affect disability progression compared with a placebo. However, Nikoo et al. concluded that RTX improved EDSS scores in NMOSD patients more efficiently than AZA, which we did not observe in our meta-analysis and subgroup analyses [12]. Thus, to compare the efficacy of different medications, one should consider not only the outcome of the network plot but also the number of trials and participants as well as the evidence from direct comparisons [45]. In addition, the fact that the EDSS is more sensitive in the evaluation of active walking than visual and cognitive functions should also be considered. Therefore, using a broader range of metrics, such as the Modified Rankin Scale (mRS), may be appropriate to clarify the specific impact of different treatment modalities on disability progression in NMOSD patients [46].

As to safety outcomes, we used both total and individual AEs, including gastrointestinal intolerance, hepatotoxicity, and leukopenia, to assess the safety of the different medications. According to the outcome of SUCRA, MMF ranked first, followed by RTX, AZA, and corticosteroids, with significant differences. However, it should be considered that MMF was only included in cohort studies, which may have induced a publication bias, causing an underestimation of the AEs of MMF. In contrast, the AZA group had a higher incidence of leukopenia, hepatotoxicity, and gastrointestinal intolerance, which confirmed the results of previous meta-analyses [47, 48]. Mcleod et al. advocated that patients treated with AZA had a genetic mutation in TPMT*3C that resulted in low thiopurine methyltransferase (TPMT) levels, leading to toxicity and an increased incidence of AEs [49]. A retrospective study with the first-line therapy of NMOSD confirmed MMF as the first-line therapeutic option and added that AZA was worth considering if the side effects were tolerable [50]. Studies have pointed out that the long-term use of AZA or MMF increases the risk of hematologic malignancies [51, 52]. Note-worthily, such threats may not spring up in short-term observations and thus could have been severely underestimated as potential side effects. Therefore, the safety of AZA and MMF should be confirmed by further studies. Among monoclonal antibodies, RTX showed a low incidence in total and individual AEs, indicating that NMOSD patients tolerated RTX well and experienced only mild or moderate adverse reactions [53].

It should be noted that these treatments are highly expensive because all AQP4-IgG seropositive patients require lifelong attack prevention [54]. Eculizumab costs about US $710,000 per patient per year, and patients need to be monitored during the injection, which prohibits its use in many places [54, 55]. Considering the high cost of eculizumab, Kim et al. advocated a switch to biological treatments such as B cell depletion treatments and IL-6 receptor inhibitors [56]. Although RTX has not received regulatory approval, it is more accessible and much cheaper than other monoclonal antibodies [54]. As to satralizumab and inebilizumab, they were less effective than RTX in reducing relapse rates. Besides, they did not demonstrate fewer AEs in the RCTs [14, 17]. Thus, considering the efficacy and safety outcomes, RTX remains the optimal choice compared with other monoclonal antibodies or immunosuppressive agents. Several studies have proven the efficacy of AZA and MMF in decreasing the risk of relapse and disability progression [57, 58]. Shi et al. advocated that the combination of glucocorticoids with AZA or MMF significantly reduced the relapse rate [59]. Although AZA and MMF did not demonstrate optimal efficacy in reducing relapse rates and EDSS in our study, they will remain the main option for most patients considering the cost-effectiveness and real-world practice.

There are several limitations in our NMA. First, the results of our study are constrained by the small sample of participants receiving monoclonal antibodies such as eculizumab, tocilizumab, and satralizumab. Despite our extensive research, we retrieved only seven published RCTs to compare the efficacy and safety of monoclonal antibodies and immunosuppressive agents. Second, although we conducted a detailed subgroup analysis of AQP4-IgG-positive NMOSD patients, we did not perform a network comparison due to the lack of RCTs. Moreover, although the heterogeneity and consistency of the network have been tested, the statistical power of this relatively weak network remains limited [45], and it is susceptible to interference factors such as the primary medical level between different clinical trials.

## Conclusion

Overall, this NMA summarized the efficacy and safety of monoclonal antibodies and immunosuppressants for NMOSD. We believe that RTX and tocilizumab have the best efficacy against NMOSD, RTX has a higher safety than other monoclonal antibodies, and the safety of MMF needs to be further confirmed by long-term analyses. RTX may be the best treatment option for clinicians. All comparisons of one treatment with another should include the possible limitations of the available data, the characteristics of the patient group, and any potential uncertainties resulting from the selection of the dose or the context of the therapy. Nevertheless, monoclonal antibodies, a novel therapeutic approach, have a wide range of potential applications in the treatment of NMOSD. Therefore, we expect to see more RCTs evaluating various monoclonal antibodies in NMOSD in the future.

## Supplementary Information

Below is the link to the electronic supplementary material.

Supplementary file1 (DOCX 351104 kb)

## Data Availability

All data generated or analyzed during this study are included in this published article and its supplementary information files.
