# Supplementary material for: Different monoclonal antibodies and immunosuppressants administration in patients with neuromyelitis optica spectrum disorder: a Bayesian network meta-analysis
Source: J Neurol. 2023 Mar 8;270(6):2950–63. doi: 10.1007/s00415-023-11641-1 (PMC10188410; doi:10.1007/s00415-023-11641-1)
Supplement: Supplementary file 1 — Supplementary file1 (DOCX 351104 kb) [file 415_2023_11641_MOESM1_ESM.docx]

**Different monoclonal antibodies and immunosuppressants administration in patients with Neuromyelitis Optica Spectrum Disorder: A Bayesian Network Meta-Analysis**

Ziqian Yin^2, #^, Youjia Qiu^1, #^, Aojie Duan^1^, Ting Fang^3^, Zhouqing Chen^1, *^, Jiang Wu^1, *^, Zhong Wang^1, *^, Gang Chen

*^1^* *Department of Neurosurgery & Brain and Nerve Research Laboratory,* *The First Affiliated Hospital of Soochow University, Suzhou, Jiangsu Province, 215006, China*

*^2^ Suzhou Medical College of Soochow University, Suzhou, Jiangsu Province, 215002, China*

*^3^* *Hainan General Hospital (Hainan Affiliated Hospital of Hainan Medical University) ，Hainan Province 570311, China*

\# Ziqian Yin and Youjia Qiu contribute equally to this work.

*Corresponding author:

Jiang Wu, Department of Neurosurgery, The First Affiliated Hospital of Soochow

University, 188 Shizi Street, Suzhou, Jiangsu Province, 215006, China. Email address: szjiangwu@163.com.

Zhong Wang, Department of Neurosurgery, The First Affiliated Hospital of Soochow University, 188 Shizi Street, Suzhou, Jiangsu Province, 215006, China. Email address: wangz8761@163.com.

Zhouqing Chen, Department of Neurosurgery, The First Affiliated Hospital of

Soochow University, 188 Shizi Street, Suzhou, Jiangsu Province, 215006, China.

Email address: zqchen6@163.com.

**Table legends**

Table S1: The detailed search strategy.

Table S2: Inclusion, Exclusion criteria, Study design and Outcome assessments of the Included Studies.

**Figure legends**

Figure S1 Risk of bias assessment of NRSIs with ROBINS-I.

Figure S2 Summary of risk of bias assessment of NRSIs with ROBINS-I.

Figure S3 Forest plots of the network meta-analysis: ARR.

Figure S4 Forest plots of the network meta-analysis: relapse.

Figure S5 Forest plots of the network meta-analysis: EDSS.

Figure S6 Forest plots of the network meta-analysis: adverse events.

Figure S7 Forest plots of the network meta-analysis: gastrointestinal intolerance.

Figure S8 Forest plots of the network meta-analysis: hepatotoxicity.

Figure S9 Forest plots of the network meta-analysis: leukopenia.

Figure S10 Forest plots for the heterogeneity: ARR.

Figure S11 Forest plots for the heterogeneity: relapse.

Figure S12 Forest plots for the heterogeneity: EDSS.

Figure S13 Forest plots for the heterogeneity: adverse events.

Figure S14 Forest plots for the heterogeneity: gastrointestinal intolerance.

Figure S15 Forest plots for the heterogeneity: hepatotoxicity.

Figure S16 Forest plots for the heterogeneity: leukopenia.

Figure S17 Forest plots for the consistency: ARR.

Figure S18 Forest plots for the consistency: relapse.

Figure S19 Forest plots for the consistency: EDSS.

Figure S20: Funnel plot for ARR.

Figure S21: Funnel plot for relapse.

Figure S22: Funnel plot for EDSS.

Figure S23: Funnel plot for adverse events.

Figure S24: Funnel plot for gastrointestinal intolerance.

Figure S25: Funnel plot for hepatotoxicity.

Figure S26: Funnel plot for leukopenia.

Figure S27: Pairwise meta-analysis of efficacy: ARR, monoclonal antibody versus placebo.

Figure S28: Pairwise meta-analysis of efficacy: ARR, immunosuppressant versus corticosteroids.

Figure S29: Pairwise meta-analysis of efficacy: relapse, monoclonal antibody versus placebo.

Figure S30: Pairwise meta-analysis of efficacy: relapse, immunosuppressant versus corticosteroids.

Figure S31: Pairwise meta-analysis of efficacy: EDSS, monoclonal antibody versus placebo.

Figure S32: Pairwise meta-analysis of efficacy: EDSS, immunosuppressant versus corticosteroids.

Figure S33: Pairwise meta-analysis of safety: AEs, monoclonal antibody versus placebo.

Figure S34: Pairwise meta-analysis of safety: AEs, immunosuppressant versus corticosteroids.

Figure S35: Pairwise meta-analysis of safety: gastrointestinal intolerance, monoclonal antibody versus placebo.

Figure S36: Pairwise meta-analysis of safety: gastrointestinal intolerance, immunosuppressant versus corticosteroids.

Figure S37: Pairwise meta-analysis of safety: hepatotoxicity, immunosuppressant versus corticosteroids.

Figure S38: Pairwise meta-analysis of safety: leukopenia, immunosuppressant versus corticosteroids.

Figure S39: Sensitivity analysis of efficacy: ARR.

Figure S40: Sensitivity analysis of efficacy: relapse.

Figure S41: Sensitivity analysis of safety: AEs.

Figure S42: Sensitivity analysis of safety: hepatotoxicity.

Figure S43: Sensitivity analysis of safety: leukopenia.

Figure S44: Network of RCTs comparing different monoclonal antibodies and immunosuppressants of

NMOSD treatments.

Figure S45: Network meta-analysis results of NMOSD treatments in RCTs.

Figure S46: Cumulative probability of each intervention with specific rank for efficacy outcomes in RCTs.

Figure S47: Cumulative probability of each intervention with specific rank for safety outcomes in RCTs.

Figure S48 Forest plots of the network meta-analysis in RCTS: ARR.

Figure S49 Forest plots of the network meta-analysis in RCTS: relapse.

Figure S50 Forest plots of the network meta-analysis in RCTS: EDSS.

Figure S51 Forest plots of the network meta-analysis in RCTS: adverse events.

Figure S52 Forest plots of the network meta-analysis in RCTS: serious adverse events.

Figure S53 Forest plots for the heterogeneity in RCTs: ARR.

Figure S54 Forest plots for the heterogeneity in RCTs: relapse.

Figure S55 Forest plots for the heterogeneity in RCTs: EDSS.

Figure S56 Forest plots for the heterogeneity in RCTs: adverse events.

Figure S57 Forest plots for the heterogeneity in RCTs: serious adverse events.

Figure S58: Subgroup analysis of aquaporin-4-IgG positive: ARR, monoclonal antibody versus placebo.

Figure S59: Subgroup analysis of aquaporin-4-IgG positive: relapse, monoclonal antibody versus other treatments.

Figure S60: Subgroup analysis of aquaporin-4-IgG positive: EDSS, monoclonal antibody versus placebo.

Figure S61: Subgroup analysis of aquaporin-4-IgG positive: AE, monoclonal antibody versus placebo.

Figure S62: Subgroup analysis of aquaporin-4-IgG positive: SAE, monoclonal antibody versus placebo.

Figure S63: PRSIMA checklist

**Table S1: Detailed Search Strategy**

Search Date: 31 May 2022

**Pubmed：**

| Search | Query | Results |
| --- | --- | --- |
| #1 | " Neuromyelitis Optica "[ MeSH Terms] | 4,038 |
| #2 | (((((((((((((( (NMO Spectrum Disorder[Title/Abstract])) OR (NMO Spectrum Disorders[Title/Abstract])) OR (Neuromyelitis Optica (NMO) Spectrum Disorder[Title/Abstract])) OR (Neuromyelitis Optica Spectrum Disorders[Title/Abstract])) OR (Devic Neuromyelitis Optica[Title/Abstract])) OR (Neuromyelitis Optica[Title/Abstract])) OR (Devic[Title/Abstract])) OR (Devic's Disease[Title/Abstract])) OR (Devic Syndrome[Title/Abstract])) OR (Devic's Neuromyelitis Optica[Title/Abstract])) OR (Neuromyelitis Optica (NMO) Spectrum Disorders[Title/Abstract])) OR (neuromyelitis optica spectrum disorder[Title/Abstract])) | 5,531 |
| #3 | #1 OR #2 | 5,925 |
| #4 | " antibodies, monoclonal "[ MeSH Terms] | 269,608 |
| #5 | ((((((((((((((((((((((((((((((((( (Monoclonal Antibodies[Title/Abstract])) OR (Monoclonal Antibody[Title/Abstract])) OR (CD20 Antibody[Title/Abstract])) OR (Rituximab CD20 Antibody[Title/Abstract])) OR (Mabthera[Title/Abstract])) OR (IDEC-C2B8 Antibody[Title/Abstract])) OR (GP2013[Title/Abstract])) OR (Rituxan[Title/Abstract])) OR (ublituximab[Title/Abstract])) OR (ofatumumab[Title/Abstract])) OR (inebilizumab[Title/Abstract])) OR (eculizumab[Title/Abstract])) OR (tocilizumab[Title/Abstract])) OR (satralizumab[Title/Abstract])) OR (bevacizumab[Title/Abstract])) OR (aquaporumab[Title/Abstract])) OR (natalizumab[Title/Abstract])) OR (alemtuzumab[Title/Abstract])) OR (immunosuppressive drug[Title/Abstract])) OR (immunosuppression[Title/Abstract])) OR (Mycophenolate Mofetil[Title/Abstract])) OR (Mofetil, Mycophenolate[Title/Abstract])) OR (Mycophenolic Acid[Title/Abstract])) OR (Mycophenolate Sodium[Title/Abstract])) OR (Myfortic[Title/Abstract])) OR (Mycophenolate Mofetil Hydrochloride[Title/Abstract])) OR (Mofetil Hydrochloride[Title/Abstract])) OR (Mycophenolate[Title/Abstract])) OR (azathioprine sodium[Title/Abstract])) OR (azathioprine[Title/Abstract])) OR (tacrolimus[Title/Abstract])) OR (Cyclosporine[Title/Abstract])) OR (ciclosporin[Title/Abstract]))) | 353,929 |
| #6 | #4 OR #5 | 484,942 |
| #7 | #3 AND #6 | **831** |

**Embass：**

| Search | Query | Results |
| --- | --- | --- |
| #1 | 'neuromyelitis optica'/exp | 11,497 |
| #2 | 'nmo spectrum disorder':ab,ti | 428 |
| #3 | 'nmo spectrum disorders':ab,ti | 423 |
| #4 | 'neuromyelitis optica (nmo) spectrum disorder':ab,ti | 57 |
| #5 | 'neuromyelitis optica spectrum disorders':ab,ti | 1,833 |
| #6 | 'devic neuromyelitis optica':ab,ti | 16 |
| #7 | 'neuromyelitis optica':ab,ti | 9,683 |
| #8 | 'devic':ab,ti | 806 |
| #9 | 'devic disease':ab,ti | 86 |
| #10 | 'devic syndrome':ab,ti | 45 |
| #11 | 'neuromyelitis optica (nmo) spectrum disorders':ab,ti | 45 |
| #12 | 'neuromyelitis optica spectrum disorder':ab,ti | 2,779 |
| #13 | #1 OR #2 OR #3 OR #4 OR #5 OR #6 OR #7 OR #8 OR #9 OR #10 OR #11 OR #12 | 12,396 |
| #14 | 'antibodies, monoclonal'/exp | 711,853 |
| #15 | 'monoclonal antibodies':ab,ti | 123,545 |
| #16 | 'monoclonal antibody':ab,ti | 144,369 |
| #17 | 'cd20 antibody':ab,ti | 2,838 |
| #18 | 'rituximab cd20 antibody':ab,ti | 6 |
| #19 | 'mabthera':ab,ti | 420 |
| #20 | 'idec-c2b8 antibody':ab,ti | 4 |
| #21 | 'gp2013':ab,ti | 34 |
| #22 | 'rituxan':ab,ti | 645 |
| #23 | 'ublituximab':ab,ti | 167 |
| #24 | 'ofatumumab':ab,ti | 1513 |
| #25 | 'inebilizumab':ab,ti | 121 |
| #26 | 'eculizumab':ab,ti | 4,706 |
| #27 | 'satralizumab':ab,ti | 132 |
| #28 | 'tocilizumab':ab,ti | 12,055 |
| #29 | 'bevacizumab':ab,ti | 34,152 |
| #30 | 'aquaporumab':ab,ti | 16 |
| #31 | 'natalizumab':ab,ti | 6,455 |
| #32 | 'alemtuzumab':ab,ti | 7,325 |
| #33 | 'immunosuppressive drug':ab,ti | 4,667 |
| #34 | 'immunosuppression':ab,ti | 102,473 |
| #35 | 'mycophenolate mofetil':ab,ti | 20,973 |
| #36 | 'mofetil, mycophenolate':ab,ti | 174 |
| #37 | 'mycophenolic acid':ab,ti | 5,171 |
| #38 | 'mycophenolate sodium':ab,ti | 975 |
| #39 | 'myfortic':ab,ti | 216 |
| #40 | 'mycophenolate mofetil hydrochloride':ab,ti | 3 |
| #41 | 'mofetil hydrochloride':ab,ti | 3 |
| #42 | 'mycophenolate':ab,ti | 27,734 |
| #43 | 'azathioprine sodium':ab,ti | 16 |
| #44 | 'azathioprine':ab,ti | 29,747 |
| #45 | 'tacrolimus':ab,ti | 37,550 |
| #46 | 'cyclosporine':ab,ti | 47,853 |
| #47 | 'ciclosporin':ab,ti | 3,795 |
| #48 | #14 OR #15 OR #16 OR #17 OR #18 OR #19 OR #20 OR #21 OR #22 OR #23 OR #24 OR #25 OR #26 OR #27 OR #28 OR #29 OR #30 OR #31 OR #32 OR #33 OR #34 OR #35 OR #36 OR #37 OR #38 OR #39 OR #40 OR #41 OR #42 OR #43 OR #44 OR #45 OR #46 OR #47 | 945,438 |
| #49 | #13 AND #48 | **3,123** |

**Cochrane:**

| Search | Query | Results |
| --- | --- | --- |
| #1 | MeSH descriptor: [Neuromyelitis Optica] explode all trees | 53 |
| #2 | (NMO Spectrum Disorder):ti,ab,kw OR (NMO Spectrum Disorders):ti,ab,kw OR (Neuromyelitis Optica (NMO) Spectrum Disorder):ti,ab,kw OR (Neuromyelitis Optica):ti,ab,kw OR (Neuromyelitis Optica Spectrum Disorders):ti,ab,kw OR (Devic's Disease):ti,ab,kw OR (neuromyelitis optica spectrum disorder):ti,ab,kw OR (Devic Neuromyelitis Optica):ti,ab,kw OR (Devic):ti,ab,kw OR (Devic Syndrome):ti,ab,kw OR (Neuromyelitis Optica (NMO) Spectrum Disorders):ti,ab,kw OR (Devic's Neuromyelitis Optica):ti,ab,kw | 298 |
| #3 | #1 or #2 | 298 |
| #4 | MeSH descriptor: [Antibodies, Monoclonal] explode all trees | 15929 |
| #5 | (Monoclonal Antibodies):ti,ab,kw OR (Monoclonal Antibody):ti,ab,kw OR (CD20 Antibody):ti,ab,kw OR (Rituximab CD20 Antibody):ti,ab,kw OR (Mabthera):ti,ab,kw OR (IDEC-C2B8 Antibody):ti,ab,kw OR (GP2013):ti,ab,kw OR (Rituxan):ti,ab,kw OR (ublituximab):ti,ab,kw OR (ofatumumab):ti,ab,kw OR (inebilizumab):ti,ab,kw OR (eculizumab):ti,ab,kw OR (tocilizumab):ti,ab,kw OR (satralizumab):ti,ab,kw OR (bevacizumab):ti,ab,kw OR (aquaporumab):ti,ab,kw OR (natalizumab):ti,ab,kw OR (alemtuzumab):ti,ab,kw OR (immunosuppressive drug):ti,ab,kw OR (immunosuppression):ti,ab,kw OR (Mycophenolate Mofetil):ti,ab,kw OR (Mofetil, Mycophenolate):ti,ab,kw OR (Mycophenolic Acid):ti,ab,kw OR (Mycophenolate Sodium):ti,ab,kw OR (Myfortic):ti,ab,kw OR (Mycophenolate Mofetil Hydrochloride):ti,ab,kw OR (Mofetil Hydrochloride):ti,ab,kw OR (azathioprine sodium):ti,ab,kw OR (Mycophenolate):ti,ab,kw OR (azathioprine):ti,ab,kw OR (tacrolimus):ti,ab,kw OR (Cyclosporine):ti,ab,kw OR (ciclosporin):ti,ab,kw | 46344 |
| #6 | #4 or #5 | 50210 |
| #7 | #3 and #6 | **195** |

**Table S2: Inclusion, exclusion criteria, study design and outcome assessments of the included studies**

| **Trials** | **Bruce A C Cree et al 2019(1)** |
| --- | --- |
| ***Inclusion*** ***Criteria*** | Patients who are older over 18 years with a diagnosis of NMOSD, at least one relapse during the preceding year, and an entry score of 0 to 5.0 on the EDSS. an Expanded Disability Status Scale (EDSS) score of 8·0 or less, and a history of either at least one attack requiring rescue therapy (intravenous cortico­steroids, intravenous immunoglobulin, plasma exchange, or a combination of these therapies) during the year before screening or at least two attacks requiring rescue therapy in the 2 years before screening. Patients who were AQP4­IgG­seropositive and AQP4­IgG­seronegative were eligible; however, patients who were seronegative also needed to meet the criteria described by Wingerchuk and colleagues(2). |
| ***Exclusion Criteria*** | NA |
| ***Study Design*** | Eligible participants were randomly allocated (3:1) to receive 300 mg intravenous inebilizumab or placebo after a screening period (≤56 days). Randomisation was done with a central interactive voice system and interactive web response system, and a permuted block randomisation scheme. |
| ***Efficacy Outcomes*** | The change of annualized rate of relapse (ARR). |
| ***Safety Outcomes*** | Adverse events, serious adverse events, the number of patients who relapsed, the number of patients with drug discontinuation, the number of patients with gastrointestinal intolerance. |

| **Trials** | **Chao Zhang et al 2019(3)** |
| --- | --- |
| ***Inclusion Criteria*** | Eligible patients were adults (≥18 years) with highly relapsing NMOSD diagnosed according to 2015 International Panel for Neuromyelitis Optica Diagnosis criteria(4), who had an Expanded Disability Status Scale (EDSS) score of 7·5 or lower, and a history of at least two clinical relapses during the previous 12 months or three relapses during the previous 24 months, with at least one relapse in the previous 12 months. |
| ***Exclusion Criteria*** | Patients were excluded if they had evidence of clinically significant infection, were pregnant or planning to conceive during the trial period, had previously relapsed on azathioprine therapy, had a heterozygous or homozygous mutation in the thiopurine methyl transferase (TPMT) gene, had received rituximab or any experimental B-cell-depleting drug in the 6 months before screening, or presented with a percentage of CD19-positive B cells in peripheral blood mononuclear cells that was higher than 1%. |
| ***Study Design*** | Eligible patients were randomly assigned (1:1) to receive either tocilizumab or azathioprine for 60 weeks. Randomisation was done centrally by an independent biostatistician at Tianjin Medical University (Tianjin, China) using computer-generated randomisation with permuted block sizes of four and was overseen by the chair of the Statistician Panel. |
| ***Efficacy Outcomes*** | NA |
| ***Safety Outcomes*** | Adverse events, serious adverse events, the number of patients who relapsed, the number of patients with drug discontinuation, the number of patients with gastrointestinal intolerance, the number of patients with hepatotoxicity. |

| **Trials** | **T. Yamamura et al 2019(5)** |
| --- | --- |
| ***Inclusion Criteria*** | Eligible patients were adolescents or adults (12 to 74 years of age) who had AQP4-IgG–seropositive or AQP4-IgG–seronegative neuromyelitis optica as defined by published criteria24 or who had AQP4-IgG–seropositive NMOSD at screening with idiopathic single or recurrent events of longitudinally extensive myelitis (≥3 vertebral-segment spinal cord lesions on magnetic resonance imaging) or recurrent or simultaneous optic neuritis in both eyes.4 The percentage of AQP4-IgG–seronegative patients was limited to approximately 30% of the total population of adults (those 18 to 74 years of age) in the trial. Patients were required to have had at least two relapses in the 2 years before screening, with at least one relapse occurring in the previous 12 months. To be eligible, patients had to have an Expanded Disability Status Scale (EDSS) score of 0 to 6.5 at screening (on a scale from 0 to 10, with a score of 0 representing no disability and a score of 10 representing death), and the dose of permitted baseline treatments must have remained stable for 8 weeks before baseline. |
| ***Exclusion Criteria*** | Key exclusion criteria were previous treatment with any agent targeting the interleukin-6 pathway, alemtuzumab, total-body irradiation, or bone marrow transplantation at any time; the use of eculizumab, belimumab, or multiple sclerosis disease–modifying treatment within 6 months before baseline; or the use of anti-CD4 agents, cladribine, or mitoxantrone within 2 years before baseline. |
| ***Study Design*** | Eligible patients were randomly assigned (1:1) to receive either tocilizumab or azathioprine for 60 weeks. Randomisation was done centrally by an independent biostatistician at Tianjin Medical University (Tianjin, China) using computer-generated randomisation with permuted block sizes of four and was overseen by the chair of the Statistician Panel. |
| ***Efficacy Outcomes*** | The change of annualized rate of relapse (ARR), and the change of EDSS socre |
| ***Safety Outcomes*** | Adverse events, serious adverse events, the number of patients who relapsed, the number of patients with drug discontinuation. |

| **Trials** | **Anthony Traboulsee et al 2020(6)** |
| --- | --- |
| ***Inclusion Criteria*** | Eligible participants were adults (aged 18–74 years) who had either AQP4­IgG seropositive or seronegative neuromyelitis optica using the 2006 Wingerchuk criteria(2), or AQP4­IgG seropositive NMOSD with either single or recurrent events of longitudinally extensive myelitis (≥3 vertebral segment spinal cord MRI lesions) or optic neuritis(7). The term NMOSD is used throughout this paper to refer to both groups of patients, in accord ance with 2015 guidelines(4). The number of AQP4­IgG seronegative patients was limited to about 30% of the total population of the study (additional enrolment for AQP4­IgG seronegative patients was stopped after Dec 2, 2016, except for patients under screening at that time, when 64 patients had already been randomly assigned). Patients were required to have clinical evidence of at least one documented attack in the 12 months before screening and a score of 6·5 or less on the Expanded Disability Status Scale (EDSS). |
| ***Exclusion Criteria*** | Exclusion criteria included clinical relapse 30 days or fewer before baseline; any prior treatment with any agent targeting the IL­6 inhibition pathway, alemtuzumab treatment, total body irradiation, or bone marrow transplantation; treatment in the past 6 months with an anti­B­lymphocyte antigen CD20, eculizumab, anti­B-lymphocyte stimulator, or any other multiple sclerosis disease­modifying treatment; treatment in the past 2 years with an anti­T­cell sur face glycoprotein CD4, cladribine, cyclophosphamide, or mitoxantrone; or treatment with any other investigational drug within 3 months prior to baseline. |
| ***Study Design*** | Participants were randomly assigned 2:1 to receive satralizumab 120 mg or placebo subcutaneously during the double­blind period using Interactive Web Response and Voice Response Systems, which assigned patients to a treatment group using the pre­defined randomisation list. Study treatments (satralizumab and placebo) were supplied by Chugai Pharmaceutical (Tokyo, Japan) in identical vials and were identical in packaging, labelling, schedule of administration, and appearance. The 2:1 ratio was selected to decrease the probability of a patient being assigned placebo. Randomisation was stratified by previous therapy for relapse prevention (B­cell depleting therapy vs immunosuppressants or other) and the nature of the most recent attack in the year before screening (patient’s first clini cal attack vs relapse). Administration of study treatment occurred on the same day as randomisation. |
| ***Efficacy Outcomes*** | The change of annualized rate of relapse (ARR), and the change of EDSS socre |
| ***Safety Outcomes*** | Adverse events, serious adverse events, the number of patients who relapsed, the number of patients with drug discontinuation, the number of patients with gastrointestinal intolerance. |

| **Trials** | **Zahra Nikoo et al 2017(8)** |
| --- | --- |
| ***Inclusion Criteria*** | The inclusion criteria were defined as: (1) a diagnosis of NMOSD based on the international consensus diagnostic criteria in 2015(4), (2) age between 18 and 50 years, (3) expanded disability status scale (EDSS) between 0 and 7. |
| ***Exclusion Crituximaberia*** | The exclusion crituximaberia were defined as: (1) pregnancy or lactation during the study, (2) decision to leave the study, (3) lack of cooperation for follow-up, (4) severe side effects of the administered medication, (5) treatment with other immunosuppressant medications (including but not limited to mycophenolate mofetil, cyclophosphamide, methotrexate) within 2 months before intervention, (6) taking any other immunosuppressant or other type of medication (including herbal drugs) without permission of the physician during the study, (7) presence of other autoimmune diseases (including but not limited to Behcet disease, systemic lupus erythematosus, rheumatoid arthrituximabis), (8) presence of liver disorders, (9) presence of hematologic disorders, (10) presence of heart failure, (11) receipt of a live vaccine within 4 weeks prior to intervention, (12) previous treatment with AZA or rituximab, (13) history of human immunodeficiency virus (HIV), hepatitis B or hepatitis C, and (14) history of severe allergic or anaphylactic reaction to monoclonal antibodies. |
| ***Study Design*** | Patients were randomly divided into two groups of AZA and rituximab by simple randomization. In order to randomize patients, we assigned a random number to each participant using Microsoft Excel function to generate random numbers. Then, patients with even numbers were allocated to AZA group and those with odd numbers were allocated to rituximab group. |
| ***Efficacy Outcomes*** | The change of annualized rate of relapse (ARR), and the change of EDSS socre. |
| ***Safety Outcomes*** | Adverse events, serious adverse events, the number of patients who relapsed, the number of patients with drug discontinuation, the number of patients with gastrointestinal intolerance, the number of patients with hepatotoxicity. |

| **Trials** | **S.J. Pittock et al 2019(9)** |
| --- | --- |
| ***Inclusion Criteria*** | Eligibility criteria included an age of at least 18 years; a diagnosis of neuromyelitis optica or NMOSD, according to 2006(2) or 2007(7) criteria, respectively; AQP4-IgG–seropositive status, as confirmed with the use of a commercially available cell-binding kit assay (Euroimmun) analyzed centrally at Mayo Medical Laboratories; a history of at least two relapses during the previous 12 months or three relapses during the previous 24 months, at least one of which had occurred within the previous 12 months; and a score of 7 or less on the EDSS(10). Patients who were receiving immunosuppressive therapies for relapse prevention were eligible for inclusion if they were receiving stable-dose regimens. |
| ***Exclusion Criteria*** | Exclusion criteria included treatment with mitoxantrone or rituximab during the previous 3 months, the receipt of intravenous immune globulin during the previous 3 weeks, the receipt of prednisone doses greater than 20 mg per day or the equivalent for other glucocorticoids at screening, and unresolved meningococcal disease or systemic bacterial or other infection considered to be clinically significant or not treated with appropriate antibiotics. |
| ***Study Design*** | At baseline (day 1), we randomly assigned patients in a 2:1 ratio to receive either eculizumab or placebo. The patients were stratified across sites according to the score on the Expanded Disability Status Scale (EDSS) on day 1 (≤ 2.0 or 2.5 to 7 .0 on a scale ranging from 0 [no disability] to 10 [death]) and the use of concomitant immunosuppressive therapy (no previous immunosuppressive therapy, except for glucocorticoids alone; continuing the same immunosuppressive therapy after the last relapse before screening, although doses may have changed; or receiving new immunosuppressive therapy or discontinuing an existing therapy after the last relapse). |
| ***Efficacy Outcomes*** | The change of annualized rate of relapse (ARR), and the change of EDSS socre. |
| ***Safety Outcomes*** | Adverse events, serious adverse events, the number of patients who relapsed, the number of patients with drug discontinuation, the number of patients with gastrointestinal intolerance. |

| **Trials** | **Masayuki Tahara et al 2020(11)** |
| --- | --- |
| ***Inclusion Criteria*** | Patients aged 16–80 years who were seropositive for AQP4 antibody (including seronegative individuals who had previously been seropositive), had a history of either optic neuritis or myelitis, were receiving oral steroids (dose ≥5 mg/day, and the dose did not change by >10% over the past 3 months before registration), had an Expanded Disability Status Scale (EDSS) score22 of 7·0 or less, and were neurologically stable. |
| ***Exclusion Criteria*** | They excluded patients who were receiving oral corticosteroid drugs (dose>30 mg/day, con verted to equivalent prednisolone dose) or who were treated with oral immunosuppressive agents other than steroids (appendix pp 4–5). |
| ***Study Design*** | They provisionally registered participants who gave informed consent at visit 1 by electronic data capture (VIEDOC; Pharma Consulting Group Japan KK, Tokyo, Japan). After exclusion of individuals who did not meet inclusion criteria, definitive registration took place at visit 2 and participants were assigned either rituximab or placebo (1:1) by dynamic random allocation, with stratification according to the number of relapses in the 2 years before assignment (one or more vs none) and oral steroid dose (converted to equivalent dose of prednisolone) at registration (5–10 mg/day, 11–20 mg/day, or 21–30 mg/day). |
| ***Efficacy Outcomes*** | The change of annualized rate of relapse (ARR), and the change of EDSS socre. |
| ***Safety Outcomes*** | Adverse events, serious adverse events, the number of patients who relapsed, the number of patients with gastrointestinal intolerance. |

| **Trials** | **Ziyan Shi et al 2020(12)** |
| --- | --- |
| ***Inclusion Criteria*** | The inclusion criteria were as follows: (1) definite NMO or NMOSD, (2) older than 16 years old,  and (3) receiving MMF, AZA, or corticosteroids (CS) as maintenance treatments. Patients with no immunotherapies (NIT) were also included for comparison. |
| ***Exclusion Criteria*** | Exclusion criteria included the following: (1) use of other immunotherapies during the previous 3 months; (2) complication with malignancies and/or other serious systemic disease; (3) free from relapse in the past 2 years before enrollment; (4) lost to follow-up after first visit; (5) irregular therapies (intermittent treatments and/or reduction of dosages); (6) treatment duration less than 12 months; (7) rituximab and other second-line immunosuppressants, including methotrexate, mitoxantrone, tacrolimus, cyclophosphamide, and cyclosporine, were not included in this study because of the small sample sizes. |
| ***Study Design*** | The MMF and AZA groups were defined as patients continuously taking MMF (1000–1500 mg/day) or AZA (100–150 mg/day), respectively, for at least 12 months. These two groups were initially treated with low doses of prednisone up to 6 months and routinely monitored for blood indicators, including blood cell counts, transaminase levels, and indicators of renal function. In addition, patients who took AZA were tested for the thiopurine methyltransferase (TPMT) genotype before treatment. It seems that most of our patients with NMOSD preferred to take MMF than AZA because of concerns about adverse effects of AZA(13). |
| ***Efficacy Outcomes*** | NA |
| ***Safety Outcomes*** | The number of patients who relapsed. |

| **Trials** | **Yan Xu et al 2016(14)** |
| --- | --- |
| ***Inclusion Criteria*** | Patients with NMOSD who all met the 2015 NMOSD diagnostic criteria(4). |
| ***Exclusion Criteria*** | Excluding 80 patients who previously received immunosuppressive agents (including CTX, methotrexate, AZA, MMF, mitoxantrone and rituximab) and 30 patients who declined to receive treatments at enrollment |
| ***Study Design*** | One hundred and nineteen patients were prescribed AZA as their initial immunosuppressive agent, which was started at 50 mg daily and then increased to 100 mg daily 4 weeks later. Thirty-eight patients were prescribed MMF as their initial immunosuppressive agent with starting and maintenance doses of 1500 mg/day for adults and 600 mg/m2 twice daily for children (no N 1500 mg/day). Forty-one patients were prescribed CTX as their initial immunosuppressive agent, and they received intravenous (iv) CTX at a dose of 400 mg weekly for 30 weeks, followed by and then AZA at 100 mg daily. All the patients were co-treated with oral prednisone, starting at 1 mg/kg daily (usually 60 mg/day) with a 5-mg dose reduction every week until a dose of 30 mg/day was attained, which was then maintained for 4 weeks. Then, a more gradual dose tapering process was initiated with a 5-mg reduction every 2 weeks, followed by a switch to 7.5 mg/day for long-term maintenance. In the acute relapsing stage, high-dose iv methylprednisolone was administered at a dose of 1000 mg/day for 5 consecutive days, which was then converted to oral prednisone. |
| ***Efficacy Outcomes*** | The change of annualized rate of relapse (ARR), and the change of EDSS socre. |
| ***Safety Outcomes*** | Adverse events, the number of patients with drug discontinuation, the number of patients with gastrointestinal intolerance, the number of patients with hepatotoxicity, the number of patients with leukopenia. |

| **Trials** | **Yang Yang et al 2018(15)** |
| --- | --- |
| ***Inclusion Criteria*** | Patients who were enrolled in our study fulfilled the following criteria: 1) all patients met the revised 2015 diagnostic criteria for NMOSD with AQP4-IgG. 2) EDSS score ranged from 2.0 to 8.5 in all included patients. 3) Other diseases, such as multiple sclerosis (MS), acute disseminated encephalomyelitis (ADEM), systemic lupus erythematosus (SLE), paraneoplastic tumor and related diseases, were excluded. 4) Patients who had received other immunosuppressive agents before the study, such as cyclophosphamide, methotrexate, tacrolimus and cyclosporin, were excluded. 5) Switching to any other immunosuppressors during the follow-up was also an exclusion criterion. 6) Patients were included in our study only when they received AZA, MMF or reduced dosage of RITUXIMAB for at least 6 months. |
| ***Exclusion Criteria*** | NA |
| ***Study Design*** | On the basis of oral prednisolone (1 mg/kg/d), patients were cotreated with different immunosuppressors randomly, after being informed of the potential efficacy, side-effects and expenses. Before the three agents were prescribed, laboratory assessments were performed to measure baseline blood cell counts, transaminase levels and renal function. Drugs were administrated only if the results of these tests were normal. In AZA group, patients began taking AZA (trade name: Imuran) with 50 mg/d and then increased to 2 mg/kg/d 2 to 4 weeks later. In MMF group, patients were given MMF (trade name: Cycopin) with starting and maintenance doses of 1000 mg/d. In RITUXIMAB group, patients were treated with 100 mg RITUXIMAB (trade name: Mabthera) which was dissolved in 250 ml of normal saline, intravenous infusion for 3 to 4 h, once per week for 4 consecutive weeks (defined as one cycle). In order to prevent anaphylactic reaction, 10 mg dexamethasone and 25 mg promethazine were administrated before. During the treatment, an ECG monitor was used to supervise patients' vital signs. CD19 + B-cell counts were monitored when our researchers visited patients at regular time and RITUXIMAB was reinjected when CD19 + B-cell counts exceeded 1%. |
| ***Efficacy Outcomes*** | The change of annualized rate of relapse (ARR), and the change of EDSS socre. |
| ***Safety Outcomes*** | Adverse events, the number of patients who relapsed, the number of patients with drug discontinuation, the number of patients with gastrointestinal intolerance, the number of patients with hepatotoxicity, the number of patients with leukopenia. |

| **Trials** | **Rui Li et al 2022(16)** |
| --- | --- |
| ***Inclusion Criteria*** | Patients with NMOSD who were selected according to the Wingerchuk 2015 diagnostic criteria(4) from January 2016 to December 2020. |
| ***Exclusion Criteria*** | NA |
| ***Study Design*** | Data are collected retrospectively at first visit and prospectively whenever the patient returns for scheduled (at least once yearly) follow-up or unscheduled visits. An independent trained evaluator (Y .W.) recorded and cleaned these data at every admission and medical visit. Regular outpatient follow-up included obtaining information on demographic data, serum AQ P4-IgG status, NMOSD attacks, long-term treatment, and Expanded Disability Status Scale (EDSS) scores. Serum AQP4 -IgG  were tested using aquaporin 4-transfected cells from a commercial sampling kit (EUROIMMUN AG, Lübeck, Germany), according to the manufacturer’s instructions. |
| ***Efficacy Outcomes*** | NA |
| ***Safety Outcomes*** | The number of patients who relapsed. |

| **Trials** | **H. Chen et al 2016(13)** |
| --- | --- |
| ***Inclusion Criteria*** | They included only patients with NMO and NMOSD who received AZA or MMF for at least 6 months from September 2009 to February 2016. |
| ***Exclusion Criteria*** | Patients who had received a different immunosuppressive agent before AZA or MMF, including methotrexate, mitoxantrone, tacrolimus, cyclophosphamide and cyclosporin, were excluded because of concerns regarding residual immunological effects. |
| ***Study Design*** | The only treatment for acute relapse was intravenous methylprednisolone with prednisolone that was tapered within 3 months. Because the effects of AZA and MMF exhibit a gradual onset, in patients  who could tolerate side-effects (i.e. oedema or rash), 10 mg of prednisolone was administered orally every other day to bridge the time required until the full onset of AZA and MMF effects. Prednisolone is considered a concurrent treatment when prescribed concurrently for more than 3 months after an acute NMO attack. During a stable phase, after being notified of the potential efficacy, side-effects and expenses associated with MMF and AZA, the patients themselves decided which agent to use. Before MMF and AZA were prescribed, tests were performed to determine baseline blood cell counts, transaminase levels and renal function. MMF and AZA were used only if the results of these tests were normal. |
| ***Efficacy Outcomes*** | The change of annualized rate of relapse (ARR), and the change of EDSS socre. |
| ***Safety Outcomes*** | Adverse events, the number of patients who relapsed, the number of patients with drug discontinuation, the number of patients with hepatotoxicity. |

| **Trials** | **Jie Lin et al 2022(17)** |
| --- | --- |
| ***Inclusion Criteria*** | The inclusion criteria included the following: (1) diagnosis of NMOSD following the 2015 revised criteria and (2) at least two infusions of RITUXIMAB treatment. |
| ***Exclusion Criteria*** | NA |
| ***Study Design*** | Patients were infused with RITUXIMAB at a dosage of 375 mg/m^2^ (body surface area) once during the first treatment. Phenergan and dexamethasone (5 mg i.v.) were applied half an hour before RITUXIMAB infusion to prevent infusion-related adverse effects. The percentage of CD19+ B cells in peripheral blood lymphocytes was measured before and every 2–3 months after treatment. The same RITUXIMAB regimen was repeated when the CD19+ B-cell count exceeded 1%. No non-RITUXIMAB immunosuppressants were  administrated with RITUXIMAB. Tests for immunoglobulin (Ig) A, IgG, and IgM; T lymphocyte spot (T-SPOT) test for tuberculosis infection; tests for fungus; and chest computed tomography were performed before RITUXIMAB re-treatments. None of the patients were administered concomitant immunosuppressants when initiating RITUXIMAB treatment. In the MMF group, all patients received an initial dose of 250 mg twice daily and ratcheted up 500 mg bid based on drug tolerance. Glucocorticoids was tapered after acute attack, and partial patients in this group were treated with a low dosage of glucocorticoids during follow-up. |
| ***Efficacy Outcomes*** | The change of annualized rate of relapse (ARR), and the change of EDSS socre. |
| ***Safety Outcomes*** | Adverse events, the number of patients who relapsed, the number of patients with drug discontinuation. |

| **Trials** | **Wenjuan Huang et al 2022(18)** |
| --- | --- |
| ***Inclusion Criteria*** | Patients meeting the following criteria were included: (1) diagnosed as NMOSD according to the criteria specified in 20151; (2) serum AQP4-antibody positive by cell-based assays; (3) treated with AZA, MMF or rituximab as initial preventive agents. |
| ***Exclusion Criteria*** | The exclusion criteria were: (1) incomplete or uncertain clinical or treatment data; (2) non-compliance with the immunotherapy regimen prescribed by NMOSD experts of the collaborative centers; (3) follow-up time less than 6 months. |
| ***Study Design*** | The demographics and longitudinal follow-up data were obtained from inpatient and outpatient medical files to form a data set, including age of disease onset, sex, onset location (optic nerve, spinal cord, brainstem, cerebral or mixed), date of onset attack and subsequent relapses, preventive treatment type, date of preventive treatment initiation, date and reason of therapy discontinuation, Expanded Disability Status Scale (EDSS) score before and after preventive treatment, and adverse events (AEs) during treatment. The data set was locked in April 2021. Statistical analyses were performed during April to May 2021. |
| ***Efficacy Outcomes*** | The change of annualized rate of relapse (ARR), and the change of EDSS socre. |
| ***Safety Outcomes*** | Adverse events, the number of patients with leukopenia, the number of patients with gastrointestinal intolerance, the number of patients with hepatotoxicity. |

| **Trials** | **Yang Yang et al 2022(19)** |
| --- | --- |
| ***Inclusion Criteria*** | Patients with NMOSD (all met the international consensus diagnostic criteria)(4, 20) were treated with immunosuppressive drugs and also oral steroids from January 2008 to November 2018. |
| ***Exclusion Criteria*** | The remaining 20 of 55 records in the AQP4-Ab positive group were excluded (ten patients received Chinese traditional medicine therapy, eight received only a small prednisolone dosage, and two received no other therapy). |
| ***Study Design*** | A total of 129 patients with NMOSD who were previously treated with immunosuppressive medicine and who had concomitant prednisone had received the AQP-4 antibody (AQP4-Ab) detection, including 55 patients seropositive for AQP4-Ab and 74 seronegative for AQP4-Ab. Among the 55 patients in the AQP4-Ab positive group, 14 received low-dose rituximab (500 mg infused, then 100 mg if the percentage of CD19? B cells reached 1%) (equivalent to 375 mg/m2 by drip infusion every week for 4 weeks; this regimen was based on that for treatment of B cell lymphoma)(11), 13 patients received azathioprine (2 mg/kg daily), and eight patients received MMF (1000 mg daily). Every patient remained on the same low-dosage regimen and the patients received treatment as soon as possible, and they underwent long-term follow-up after the drug treatment. |
| ***Efficacy Outcomes*** | The change of annualized rate of relapse (ARR), and the change of EDSS socre. |
| ***Safety Outcomes*** | Adverse events, serious adverse events, the number of patients with leukopenia, the number of patients who relapsed, the number of patients with hepatotoxicity. |

| **Trials** | **Meini Zhang et al 2017(21)** |
| --- | --- |
| ***Inclusion Criteria*** | All the patients matched Wingerchuk’s 2015 diagnosis criteria for NMO and NMOSD(4) and were treated with high-dose IV methylprednisolone for acute attacks in the  First College of Clinical Medicine, Shanxi Medical University, China, from February 1, 2009 to September 30 2016. |
| ***Exclusion Criteria*** | NA |
| ***Study Design*** | The AZA group patients received AZA (2 mg/kg daily) combined with oral steroid therapy (1 mg/kg body weight/day) after the first acute attack for more than 6 months because the treatment usually takes full effect after 3–6 months(22). The RX group patients’ percentage of circulating CD19+B cells among the total lymphocytes was determined before the first infusion as the baseline level. All the patients in this group were infused with RX at 100 mg (equivalent of 50–59 mg/m2), once per week for 3 consecutive weeks, and infused three times at 4-week intervals from the last infusion (the 7th week from the first infusion) After determining the CD19+B cells again, RX was reinfused at 100 mg if the percentage of CD19+B cells reached C1%(23). |
| ***Efficacy Outcomes*** | The change of annualized rate of relapse (ARR), and the change of EDSS socre. |
| ***Safety Outcomes*** | Adverse events, serious adverse events, the number of patients with leukopenia, the number of patients who relapsed, the number of patients with hepatotoxicity. |

| **Trials** | **Shugang Cao et al 2021(24)** |
| --- | --- |
| ***Inclusion Criteria*** | The inclusion criteria were as follows: patients who fulfilled the IPND 2015 diagnostic criteria for NMOSD(4) and were regularly followed up for at least 6 months and patients who regularly received treatment with immunosuppressants (AZA, MMF, and mRTX) and were followed up for at least 3 months after receiving immunosuppressant therapy. |
| ***Exclusion Criteria*** | The exclusion criteria were as follows: patients negative for aquaporin-4 antibody (AQP4-Ab) and initially suspected of having NMOSD but later diagnosed with other diseases; patients positive for myelin oligodendrocyte glycoprotein antibody (MOG-Ab); patients treated only with steroids or non-first-line immunosuppressants in the remission phase; patients who did not regularly use, spontaneously discontinued or switched immunosuppressants (due to adverse reactions, pregnancy, dementia, and mental disorder); patients diagnosed with or highly suspected of having malignant tumors; patients who could not use immunosuppressive drugs due to recent hepatitis or active tuberculosis; patients treated with immunosuppressants for less than 3 months; and patients lost to follow-up during the retrospective case collection period. |
| ***Study Design*** | Immunosuppressants were administered in patients with NMOSD after the presence of tumors, hepatitis, or active tuberculosis was excluded. Patients were assigned to 3 groups based on immunosuppressive drug use: AZA, MMF, and mRTX groups. For the AZA group, AZA (2–3 mg/kg/d oral, 2–3 times a day) was administered in the remission phase; to guide administration, thiopurine methyltransferase (TPMT) activity was regularly assessed, or related gene assays were  performed before AZA treatment. For the MMF group, MMF (1000–1500 mg/d oral, twice a day) was administered in the remission phase. For the mRTX group, the rituximab regimen (100 mg infused on the first day and 500 mg infused on the next day,) was applied, with repeated assessment of peripheral blood B lymphocyte subsets; reinfusion of the mRTX regimen was given once when the percentage of total CD19+ B cells reached 3%. Corticosteroids were tapered and discontinued within 3 months of mRTX administration, and the mRTX regimen was used as monotherapy in patients with NMOSD(25). |
| ***Efficacy Outcomes*** | NA |
| ***Safety Outcomes*** | Adverse events, serious adverse events, the number of patients with leukopenia, the number of patients who relapsed, the number of patients with hepatotoxicity. |

| **Trials** | **Liang Wang et al 2021(26)** |
| --- | --- |
| ***Inclusion Criteria*** | The inclusion criteria were: (1) changing from another immunosuppressant or not having been under immunosuppressive treatment before; (2) meeting the diagnostic criteria of NMOSD established by the International Panel in 2015; (3) no less than half a year of TAC treatment course; (4) age ≥ 18 years. Demographics and baseline clinical data were collected, including gender, age at disease onset, disease duration, relapsing or monophasic course, TAC treatment dose and duration, AQP4-ab status, patients with combined prednisone, concomitant autoantibody status, onset location and previous immunotherapy. |
| ***Exclusion Criteria*** | NA |
| ***Study Design*** | This is a single-center retrospective observational cohort study. Patients with NMOSD under low-dose TAC treatment (1-3 mg/day) were retrospectively collected in the Department of Neurology of Hua-shan Hospital during the past six years. |
| ***Efficacy Outcomes*** | NA |
| ***Safety Outcomes*** | Adverse events, the number of patients with leukopenia, the number of patients who relapsed, the number of patients with hepatotoxicity. |

| **Trials** | **Chen Du et al 2021(27)** |
| --- | --- |
| ***Inclusion Criteria*** | The criteria for inclusion were that 1) patients were experiencing acute attacks, where 2) a moderate-to-severe myelitis attack was defined as having any one of the following: EDSS ≥ 3.5 for the first-ever myelitis(10) at least a two-point increase of the EDSS score if the previous score was ≤ 5.0, or at least a one-point worsening of their EDSS score if the previous score was ≥5.5. |
| ***Exclusion Criteria*** | The exclusion criteria were the patients who (1) used B-cell depleting agents and complement inhibitors during attacks and remission, (2) initiated tocilizumab therapy after acute attacks, and (3) lost follow-ups (4) concomitant with other diseases that may affect disability evaluation (e.g., stroke and Parkinson’s disease). |
| ***Study Design*** | HDMP or HDMP+IVIG was administered to patients to treat acute attacks. In the tocilizumab group, treatment with tocilizumab was initiated at the dose of 8 mg/kg within 2 weeks of the attack onset, followed by a routine infusion at 4-week intervals. Prednisone was gradually tapered to 10–15 mg/day for maintenance. In the prednisone group, prednisone was gradually tapered to a dose of 10–15 mg/day for attack prevention. |
| ***Efficacy Outcomes*** | NA |
| ***Safety Outcomes*** | The number of patients who relapsed. |

| **Trials** | **Maureen A. Mealy et al 2014(28)** |
| --- | --- |
| ***Inclusion Criteria*** | Institutional review board approval was obtained from the Johns Hopkins University and Mayo Clinic. Deidentified data were used; therefore, patient consent was not required. Patients were included if they were diagnosed as having NMO based on the 2006 revised NMO criteria that require occurrence of both ON and TM, along with 2 of the 3 following supportive features: (1) longitudinally extensive spinal cord lesion (≥3 vertebral segments), (2) magnetic resonance imaging of the brain nondiagnostic for MS, and (3) AQP4-IgG seropositivity(2). We defined NMOSD as TM, ON, or brainstem inflammation associated with serum AQP4-IgG.( DRUGDEX® System [Internet database]. Greenwood Village, Colorado: Thomson Reuters (Healthcare) Inc.) Only NMO and NMOSD patients who received azathioprine or mycophenolate for at least 6 months or rituximab for at least 1 month were included. Patients who were taking azathioprine, mycophenolate, or rituximab and then switched to another 1 of these 3 therapies were included if the final therapy was used for at least 6 months with azathioprine or mycophenolate or at least 1 month with rituximab regardless of duration of initial therapy. Patients who had previously been receiving immunomodulatory therapies, including glatiramer acetate, β-interferons, prednisonemono therapy, or hydroxychloroquine sulfate, were included. |
| ***Exclusion Criteria*** | Exposure to another general immunosuppressive agent, including cyclophosphamide, methotrexate, and mitoxantrone, was an exclusion criterion. Patients who were concurrently receiving more than 1 of the medications being analyzed in this analysis were also excluded. |
| ***Study Design*** | For the purpose of this analysis, azathioprine treatment was suboptimal if the relapse occurred within 6 months of drug therapy initiation or if the dosage was less than 2 mg/kg/d. Criteria for optimal dosing of azathioprine have not been established and were not standardized among centers. Suboptimal treatment with mycophenolate was defined as duration of therapy of less than 6 months or a dose that did not lower the absolute lymphocyte count to less than 1500/μL (to convert to ×109/L, multiply by 0.001) (up to 3000 mg/d). Suboptimal dosing for rituximab was defined as a duration of less than 1 month or greater than 5 months or as the presence of CD19 cells in circulation (>0.1% of total lymphocytes). |
| ***Efficacy Outcomes*** | NA |
| ***Safety Outcomes*** | The number of patients who relapsed. |

| **Trials** | **Lu Zhang et al 2021(29)** |
| --- | --- |
| ***Inclusion Criteria*** | The inclusion criteria were (1) all patients who met the NMOSD diagnostic criteria revised in 2015; (2) patients receiving AZA, MMF, or rituximab as initial immunosuppressive therapy for at least 6 months; (3) patients cooperating to complete efficacy evaluation and the follow-up; and (4) patients in remission stage. |
| ***Exclusion Criteria*** | The exclusion criteria were (1) patients with a clear diagnosis of other CNS inflammatory demyelinating diseases; (2) complication with other severe systemic disease and/or mental illness; (3) patients who accepted other immunotherapies before receiving AZA, MMF, or rituximab; and (4) patients lost to follow-up. |
| ***Study Design*** | The study is designed as a retrospective one. NMOSD patients (according to the 2015 diagnostic criteria(4) treated at the Second Hospital of Hebei Medical University between 2015 and 2019 were included. |
| ***Efficacy Outcomes*** | The change of ARR. |
| ***Safety Outcomes*** | Adverse events, the number of patients with leukopenia, the number of patients with drug discontinuation, the number of patients with gastrointestinal intolerance, the number of patients with hepatotoxicity, the number of patients who relapsed. |

| **Trials** | **In Hye Jeong et al 2015(30)** |
| --- | --- |
| ***Inclusion Criteria*** | Patients with relapsing NMO (according to the 2006 diagnostic criteria(2) or NMOSD(7) who received azathioprine, MMF, or rituximab as their initial immunosuppressive therapy were consecutively recruited from May 2005 to July 2014. |
| ***Exclusion Criteria*** | Patients with previous exposure to other immunosuppressants, such as mitoxantrone, methotrexate, and cyclophosphamide, were excluded from the present study, |
| ***Study Design*** | All clinical data were obtained by a retrospective review of medical records. |
| ***Efficacy Outcomes*** | The change of EDSS score. |
| ***Safety Outcomes*** | The number of patients who relapsed, the number of patients with leukopenia, the number of patients with drug discontinuation, the number of patients with gastrointestinal intolerance, the number of patients with hepatotoxicity. |

| **Trials** | **Nirmeen A. Kishk et al 2021(31)** |
| --- | --- |
| ***Inclusion Criteria*** | Retrospective analysis of patients’ medical records with a working diagnosis of NMO/NMOSD attending the multiple sclerosis clinic, Kasr Alainy hospital, Cairo University, between January 2013 and June 2018 was done. |
| ***Exclusion Criteria*** | NA |
| ***Study Design*** | Retrospective analysis of patients’ medical records with a working diagnosis of NMO/NMOSD attending the multiple sclerosis clinic, Kasr Alainy hospital, Cairo University, between January 2013 and June 2018 was done. |
| ***Efficacy Outcomes*** | The change of ARR. |
| ***Safety Outcomes*** | The number of patients who relapsed, the number of patients with leukopenia, the number of patients with drug discontinuation, the number of patients with gastrointestinal intolerance, the number of patients with hepatotoxicity. |

**Table S24: Inclusion, exclusion criteria, study design and outcome assessments of the included studies**

| **Trials** | **Julien Poupart et al 2020(32)** |
| --- | --- |
| ***Inclusion Criteria*** | They included patients according to the following criteria: (1) a diagnosis of NMOSD according to the Wingerchuk 2015 criteria26 and positive for anti-AQP4 or anti-MOG antibodies; (2) initiation of immunosuppressive treatment as a first-line therapy within 3 years after disease onset; and (3) at least 1 year of follow-up (whether the treatment was stopped or not). |
| ***Exclusion Criteria*** | They excluded patients with seronegative NMOSD and those who previously received immunosuppressive therapies that are specifically used for multiple sclerosis (MS) (e.g., natalizumab, fingolimod). |
| ***Study Design*** | We designed a multicenter, retrospective cohort study involving 10 French university hospitals in the setting of the French network on multiple sclerosis and NMOSD (OFSEP and NOMADMUS study groups). |
| ***Efficacy Outcomes*** | The change of ARR. |
| ***Safety Outcomes*** | The number of patients with drug discontinuation, the number of patients with gastrointestinal intolerance, the number of patients with hepatotoxicity. |

**Table S24: Inclusion, exclusion criteria, study design and outcome assessments of the included studies**

| **Trials** | **Laura Clarke et al 2022(33)** |
| --- | --- |
| ***Inclusion Criteria*** | The inclusion criteria were: (1) meet 2015 IPND criteria for a diagnosis of NMOSD; (2) positive serum AQP4 antibodies; and (3) complete data relating to relapse and treatment history. |
| ***Exclusion Criteria*** | Cases were excluded if they had an alternate diagnosis, had incomplete clinical data or had spent less than one month on therapy. Due to the wide range of dosages used, both between and within cases, concurrent use of maintenance glucocorticoid steroids was not factored into the analysis. |
| ***Study Design*** | This was a retrospective, unblinded, observational study of treatment outcomes of patients with NMOSD treated with therapies administered at the discretion of the treating physician. The study covers historical data extending prior to the discovery of AQP4 antibodies and thus includes MS disease modifying therapies in some cases. |
| ***Efficacy Outcomes*** | The change of ARR. |
| ***Safety Outcomes*** | NA |

**Figure S1 Risk of bias assessment of NRSIs with ROBINS-I.**

**Figure S2 Summary of risk of bias assessment of NRSIs with ROBINS-I.**

**Figure S3 Forest plots of the network meta-analysis: ARR.**

**Figure S4 Forest plots of the network meta-analysis: relapse.**

**Figure S5 Forest plots of the network meta-analysis: EDSS.**

**Figure S6 Forest plots of the network meta-analysis: adverse events.**

**Figure S7 Forest plots of the network meta-analysis: gastrointestinal.**

**Figure S8 Forest plots of the network meta-analysis: hepatotoxicity.**

**Figure S9 Forest plots of the network meta-analysis: Leukopenia.**

**Figure S10 Forest plots for the heterogeneity: ARR.**

**Figure S11 Forest plots for the heterogeneity: relapse.**

Figure S12 Forest plots for the heterogeneity: EDSS.

**Figure S13 Forest plots for the heterogeneity: adverse events.**

**Figure S14 Forest plots for the heterogeneity: gastrointestinal intolerance.**

**Figure S15 Forest plots for the heterogeneity: hepatotoxicity.**

**Figure S16 Forest plots for the heterogeneity: leukopenia.**

**Figure S17 Forest plots for the consistency: ARR.**

**Figure S18 Forest plots for the consistency: AE.**

**Figure S19 Forest plots for the consistency: EDSS.**

**Figure S20: Funnel plot for ARR**

**Figure S21: Funnel plot for relapse**

**Figure S22: Funnel plot for EDSS**

**Figure S23: Funnel plot for adverse events**

**Figure S24: Funnel plot for gastrointestinal intolerance**

**Figure S25: Funnel plot for hepatotoxicity**

**Figure S26: Funnel plot for leukopenia**

**Figure S27: Pairwise meta-analysis of efficacy: ARR, monoclonal antibody versus placebo.**

**Figure S28: Pairwise meta-analysis of efficacy: ARR, immunosuppressant versus corticosteroids.**

**Figure S29: Pairwise meta-analysis of efficacy: relapse, monoclonal antibody versus placebo.**

**Figure S30: Pairwise meta-analysis of efficacy: relapse, immunosuppressant versus corticosteroids.**

**Figure S31: Pairwise meta-analysis of efficacy: EDSS, monoclonal antibody versus placebo.**

**Figure S32: Pairwise meta-analysis of efficacy: EDSS, immunosuppressant versus corticosteroids.**

**Figure S33: Pairwise meta-analysis of safety: AEs, monoclonal antibody versus placebo.**

**Figure S34: Pairwise meta-analysis of safety: AEs, immunosuppressant versus corticosteroids.**

**Figure S35: Pairwise meta-analysis of safety: gastrointestinal intolerance, monoclonal antibody versus placebo.**

**Figure S36: Pairwise meta-analysis of safety: gastrointestinal intolerance, immunosuppressant versus corticosteroids.**

**Figure S37: Pairwise meta-analysis of safety: hepatotoxicity, immunosuppressant versus corticosteroids.**

**Figure S38: Pairwise meta-analysis of safety: leukopenia, immunosuppressant versus corticosteroids.**

**Figure S39: Sensitivity analysis of efficacy: ARR, monoclonal antibody versus placebo.**

**Figure S40: Sensitivity analysis of efficacy: relapse, monoclonal antibody versus placebo.**

**Figure S41: Sensitivity analysis of safety: AEs, immunosuppressant versus corticosteroids.**

**Figure S42: Sensitivity analysis of safety: hepatotoxicity, immunosuppressant versus corticosteroids.**

**Figure S43: Sensitivity analysis of safety: leukopenia.**

**Figure S44: Network of RCTs comparing different monoclonal antibodies and immunosuppressants of NMOSD treatments.**

**Figure S45: Network meta-analysis results of NMOSD treatments in RCTs.**

**Figure S46: Cumulative probability of each intervention with specific rank for efficacy outcomes in RCTs: a**. ARR. **b**. relapse. **c**. EDSS.

**Figure S47: Cumulative probability of each intervention with specific rank for safety outcomes in RCTs: a**. AE. **b**. SAE.

**Figure S48 Forest plots of the network meta-analysis in RCTS: ARR.**

**Figure S49 Forest plots of the network meta-analysis in RCTS: relapse.**

**Figure S50 Forest plots of the network meta-analysis in RCTS: EDSS.**

**Figure S51 Forest plots of the network meta-analysis in RCTS: adverse events.**

**Figure S52 Forest plots of the network meta-analysis in RCTS: serious adverse events.**

**Figure S53 Forest plots for the heterogeneity in RCTs: ARR.**

**Figure S54 Forest plots for the heterogeneity in RCTs: relapse.**

**Figure S55 Forest plots for the heterogeneity in RCTs: EDSS.**

**Figure S56 Forest plots for the heterogeneity in RCTs: adverse events.**

**Figure S57 Forest plots for the heterogeneity in RCTs: serious adverse events.**

**Figure S58: Subgroup analysis of aquaporin-4-IgG positive: ARR, monoclonal antibody versus placebo.**

**Figure S59: Subgroup analysis of aquaporin-4-IgG positive: relapse, monoclonal antibody versus other treatments.**

**Figure S60: Subgroup analysis of aquaporin-4-IgG positive: EDSS, monoclonal antibody versus placebo.**

**Figure S61: Subgroup analysis of aquaporin-4-IgG positive: AE, monoclonal antibody versus placebo.**

**Figure S62: Subgroup analysis of aquaporin-4-IgG positive: SAE, monoclonal antibody versus placebo.**

**Figure S63: PRSIMA checklist**

**Reference:**

1.	Cree BAC, Bennett JL, Kim HJ, Weinshenker BG, Pittock SJ, Wingerchuk DM, et al. Inebilizumab for the treatment of neuromyelitis optica spectrum disorder (N-MOmentum): a double-blind, randomised placebo-controlled phase 2/3 trial. Lancet. 2019;394(10206):1352-63.

2.	Wingerchuk DM, Lennon VA, Pittock SJ, Lucchinetti CF, Weinshenker BG. Revised diagnostic criteria for neuromyelitis optica. Neurology. 2006;66(10):1485-9.

3.	Zhang C, Zhang M, Qiu W, Ma H, Zhang X, Zhu Z, et al. Safety and efficacy of tocilizumab versus azathioprine in highly relapsing neuromyelitis optica spectrum disorder (TANGO): an open-label, multicentre, randomised, phase 2 trial. Lancet Neurol. 2020;19(5):391-401.

4.	Wingerchuk DM, Banwell B, Bennett JL, Cabre P, Carroll W, Chitnis T, et al. International consensus diagnostic criteria for neuromyelitis optica spectrum disorders. Neurology. 2015;85(2):177-89.

5.	Yamamura T, Kleiter I, Fujihara K, Palace J, Greenberg B, Zakrzewska-Pniewska B, et al. Trial of Satralizumab in Neuromyelitis Optica Spectrum Disorder. The New England journal of medicine. 2019;381(22):2114-24.

6.	Traboulsee A, Greenberg BM, Bennett JL, Szczechowski L, Fox E, Shkrobot S, et al. Safety and efficacy of satralizumab monotherapy in neuromyelitis optica spectrum disorder: a randomised, double-blind, multicentre, placebo-controlled phase 3 trial. Lancet Neurol. 2020;19(5):402-12.

7.	Wingerchuk DM, Lennon VA, Lucchinetti CF, Pittock SJ, Weinshenker BG. The spectrum of neuromyelitis optica. Lancet Neurol. 2007;6(9):805-15.

8.	Nikoo Z, Badihian S, Shaygannejad V, Asgari N, Ashtari F. Comparison of the efficacy of azathioprine and rituximab in neuromyelitis optica spectrum disorder: a randomized clinical trial. Journal of neurology. 2017;264(9):2003‐9.

9.	Pittock SJ, Berthele A, Fujihara K, Kim HJ, Levy M, Palace J, et al. Eculizumab in Aquaporin-4-Positive Neuromyelitis Optica Spectrum Disorder. The New England journal of medicine. 2019;381(7):614-25.

10.	Kurtzke JF. Rating neurologic impairment in multiple sclerosis: an expanded disability status scale (EDSS). Neurology. 1983;33(11):1444-52.

11.	Tahara M, Oeda T, Okada K, Kiriyama T, Ochi K, Maruyama H, et al. Safety and efficacy of rituximab in neuromyelitis optica spectrum disorders (RIN-1 study): a multicentre, randomised, double-blind, placebo-controlled trial. The lancet Neurology. 2020;19(4):298‐306.

12.	Shi Z, Du Q, Chen H, Zhang Y, Qiu Y, Zhao Z, et al. Effects of immunotherapies and prognostic predictors in neuromyelitis optica spectrum disorder: a prospective cohort study. J Neurol. 2020;267(4):913-24.

13.	Chen H, Qiu W, Zhang Q, Wang J, Shi Z, Liu J, et al. Comparisons of the efficacy and tolerability of mycophenolate mofetil and azathioprine as treatments for neuromyelitis optica and neuromyelitis optica spectrum disorder. European Journal of Neurology. 2017;24(1):219-26.

14.	Xu Y, Wang Q, Ren HT, Qiao L, Zhang Y, Fei YY, et al. Comparison of efficacy and tolerability of azathioprine, mycophenolate mofetil, and cyclophosphamide among patients with neuromyelitis optica spectrum disorder: A prospective cohort study. J Neurol Sci. 2016;370:224-8.

15.	Yang Y, Wang CJ, Wang BJ, Zeng ZL, Guo SG. Comparison of efficacy and tolerability of azathioprine, mycophenolate mofetil, and lower dosages of rituximab among patients with neuromyelitis optica spectrum disorder. J Neurol Sci. 2018;385:192-7.

16.	Li R, Li C, Huang Q, Liu Z, Chen J, Zhang B, et al. Immunosuppressant and neuromyelitis optica spectrum disorder: optimal treatment duration and risk of discontinuation. Eur J Neurol. 2022;29(9):2792-800.

17.	Lin J, Xue B, Li J, Zhu R, Pan J, Chen Z, et al. Comparison of Long-Term Use of Low Dose Rituximab and Mycophenolate Mofetil in Chinese Patients With Neuromyelitis Optica Spectrum Disorder. Front Neurol. 2022;13:891064.

18.	Huang W, Wang L, Xia J, Li W, Wang M, Yu J, et al. Efficacy and safety of azathioprine, mycophenolate mofetil, and reduced dose of rituximab in neuromyelitis optica spectrum disorder. Eur J Neurol. 2022;29(8):2343-54.

19.	Yang Y, Chen L, Wu L, Yao J, Wang N, Su X, et al. Effective Rituximab Treatment in Patients with Neuromyelitis Optica Spectrum Disorders Compared with Azathioprine and Mycophenolate. Neurology and therapy. 2022;11(1):137-49.

20.	Jacob A, Weinshenker BG, Violich I, McLinskey N, Krupp L, Fox RJ, et al. Treatment of neuromyelitis optica with rituximab: retrospective analysis of 25 patients. Archives of neurology. 2008;65(11):1443-8.

21.	Zhang M, Zhang C, Bai P, Xue H, Wang G. Effectiveness of low dose of rituximab compared with azathioprine in Chinese patients with neuromyelitis optica: an over 2-year follow-up study. Acta neurologica Belgica. 2017;117(3):695-702.

22.	Watanabe S, Misu T, Miyazawa I, Nakashima I, Shiga Y, Fujihara K, et al. Low-dose corticosteroids reduce relapses in neuromyelitis optica: a retrospective analysis. Mult Scler. 2007;13(8):968-74.

23.	Yang CS, Yang L, Li T, Zhang DQ, Jin WN, Li MS, et al. Responsiveness to reduced dosage of rituximab in Chinese patients with neuromyelitis optica. Neurology. 2013;81(8):710-3.

24.	Cao S, Yu H, Tian J, Li Y, Shen Y, Ji X, et al. Efficacy and safety of modified reduced-dose rituximab in Chinese patients with neuromyelitis optica spectrum disorder: A retrospective cohort study. J Neurol Sci. 2021;429:117616.

25.	Ciron J, Audoin B, Bourre B, Brassat D, Durand-Dubief F, Laplaud D, et al. Recommendations for the use of Rituximab in neuromyelitis optica spectrum disorders. Revue neurologique. 2018;174(4):255-64.

26.	Wang L, Tan H, Huang W, ZhangBao J, Chang X, Zhou L, et al. Low-dose tacrolimus in treating neuromyelitis optica spectrum disorder. Mult Scler Relat Disord. 2021;48:102707.

27.	Du C, Zeng P, Han JR, Zhang TX, Jia D, Shi FD, et al. Early Initiation of Tocilizumab Treatment Against Moderate-to-Severe Myelitis in Neuromyelitis Optica Spectrum Disorder. Front Immunol. 2021;12:660230.

28.	Mealy MA, Wingerchuk DM, Palace J, Greenberg BM, Levy M. Comparison of relapse and treatment failure rates among patients with neuromyelitis optica: multicenter study of treatment efficacy. JAMA neurology. 2014;71(3):324-30.

29.	Zhang L, Tian J, Dong X, Jia Z, Sun Y, Guo L, et al. Efficacy of azathioprine, mycophenolate mofetil, and rituximab in the treatment of neuromyelitis optica spectrum disorder and analysis of prognostic factors. Neurological sciences : official journal of the Italian Neurological Society and of the Italian Society of Clinical Neurophysiology. 2022;43(4):2651-8.

30.	Jeong IH, Park B, Kim SH, Hyun JW, Joo J, Kim HJ. Comparative analysis of treatment outcomes in patients with neuromyelitis optica spectrum disorder using multifaceted endpoints. Mult Scler. 2016;22(3):329-39.

31.	Kishk NA, Abdelfattah W, Shalaby NM, Shehata HS, Hassan A, Hegazy MI, et al. The aquaporin4-IgG status and how it affects the clinical features and treatment response in NMOSD patients in Egypt. BMC neurology. 2021;21(1):53.

32.	Poupart J, Giovannelli J, Deschamps R, Audoin B, Ciron J, Maillart E, et al. Evaluation of efficacy and tolerability of first-line therapies in NMOSD. Neurology. 2020;94(15):e1645-e56.

33.	Clarke L, Bukhari W, O'Gorman CM, Khalilidehkordi E, Arnett S, Woodhall M, et al. Response to treatment in NMOSD: the Australasian experience. Mult Scler Relat Disord. 2022;58:103408.
